# Atomically Precise Clusterzymes: A Programmable Optoelectronic Platform for Neuroscience

**DOI:** 10.1002/advs.202519438

**Published:** 2026-02-10

**Authors:** Si Sun, Di Liu, Sufei Zhou, Yang Wang, Hao Wang, Ziliang Zheng, Xiao‐Dong Zhang

**Affiliations:** ^1^ State Key Laboratory of Advanced Medical Materials and Devices Academy of Medical Engineering and Translational Medicine Tianjin University Tianjin 300072 China; ^2^ Tianjin Key Laboratory of Low Dimensional Materials Physics and Preparing Technology School of Science Tianjin University Tianjin 300354 China; ^3^ Laboratory of Molecular Imaging Fifth Hospital of Shanxi Medical University (Shanxi Provincial People’s Hospital) Taiyuan 030000 China

**Keywords:** atomic precision, brain computer interface, clusters, neuroscience, programmable platform

## Abstract

Atomically precise metal clusters, characterized by their well‐defined structures, have emerged as a versatile platform for energy, catalysis, and biomedicine. Building upon this foundation, the biocatalytic clusterzymes, a class of artificial enzymes with atomic‐level programmable activity and renal‐excreted properties, have successfully overcome the stability limitations of natural enzymes and biosafety concerns of conventional nanomaterials. This review systematically examines the synthesis, engineering principles, and applications of this programmable platform. First an in‐depth analysis of the strategies is provided for programming biocatalytic or enzyme‐like activity of metal clusters via atomic and ligand engineering. Meanwhile, infrared emissive metal clusters with tunable electronic structure and optical properties at the atomic level allow to achieve the pathological progression and clinical 3D visualization in deep tissue. Furthermore, semiconductor gold clusters with rich electron carriers can enhance the interface charge transfer between the metal electrode and surface molecular clusters, achieving highly sensitive neuron recording for an efficient brain computer interface. The clusters demonstrate great potential in neuroscience, including neuroinflammation, bioimaging, and neuromodulation. Finally, future challenges are outlined for the rational design and translational development of this programmable platform, poised to address complex challenges in biomedicine.

## Graphical abstract

Atomically precise clusterzymes, engineered via atomic and ligand design, serve as programmable platforms for neuroscience applications. They demonstrate tailored biocatalysis for neuroinflammation, tunable optical properties for deep‐tissue bioimaging and 3D visualization, and enhanced charge transfer for brain–computer interfaces. This review systematically examines their design principles and therapeutic potential in brain disorders.

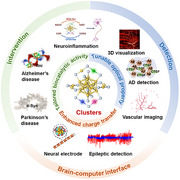

## 1 Introduction

Enzymes, the quintessential biocatalysts, orchestrate nearly all biochemical processes in living systems with extraordinary efficiency and stereochemical precision. From oxidoreductases that manage cellular redox states to hydrolases that cleave biomolecular bonds, these specialized proteins are indispensable for sustaining life. However, their exquisite adaptation to the physiological milieu renders them suboptimal for many biomedical and industrial applications, resulting from several inherent drawbacks, including expensive costs, limited stability beyond physiological environments, demanding storage conditions, and vulnerability to enzymatic degradation.^[^ ^1^, ^2^, ^3^, ^4^, ^5^, ^6^ ^]^ This gap between biological prowess and practical utility has driven a decades‐long quest to create artificial enzymes that replicate the catalytic principles of nature while overcoming these limitations.

Since Yan et al. discovered that Fe_3_O_4_ nanoparticles possess intrinsic peroxidase‐like (POD‐like) activity in 2007, a new era of nanozymes was ushered.^[^ ^7^, ^8^, ^9^, ^10^, ^11^, ^12^, ^13^, ^14^, ^15^, ^16^ ^]^ Nanozymes offer superior stability but suffer from poor biocatalytic activity and selectivity due to heterogeneous active sites.^[^ ^2^, ^17^, ^18^, ^19^, ^20^, ^21^, ^22^, ^23^, ^24^, ^25^, ^26^, ^27^, ^28^ ^]^ A major advance arrived in 2011 with the conceptualization of single‐atom nanozyme, which achieved high activity via atomic dispersion, yet their precise coordination and long‐term biosafety remain challenged.^[^ ^17^, ^29^, ^30^, ^31^ ^]^ These unresolved challenges highlight the critical need for a next‐generation platform with atomic‐level programmable activity and biosafety, where the entire biocatalytic structure is unequivocally defined and programmable.^[^ ^16^, ^32^, ^33^ ^]^

The advent of atomically precise metal clusters presents a solution to these challenges.^[^ ^34^, ^35^, ^36^, ^37^ ^]^ Metal clusters, composed of a few to tens of metal atoms and can be stabilized by a shell of protecting ligands, are typically between 2 and 5 nm and possess a determinate atomic structure.^[^ ^38^, ^39^ ^]^ This inherent atomic‐level precision provides an unprecedented platform for establishing definitive structure–property relationships, thereby addressing the critical challenge of heterogeneity in nanozymes.^[^ ^40^, ^41^, ^42^, ^43^, ^44^, ^45^, ^46^ ^]^

**Figure 1:**
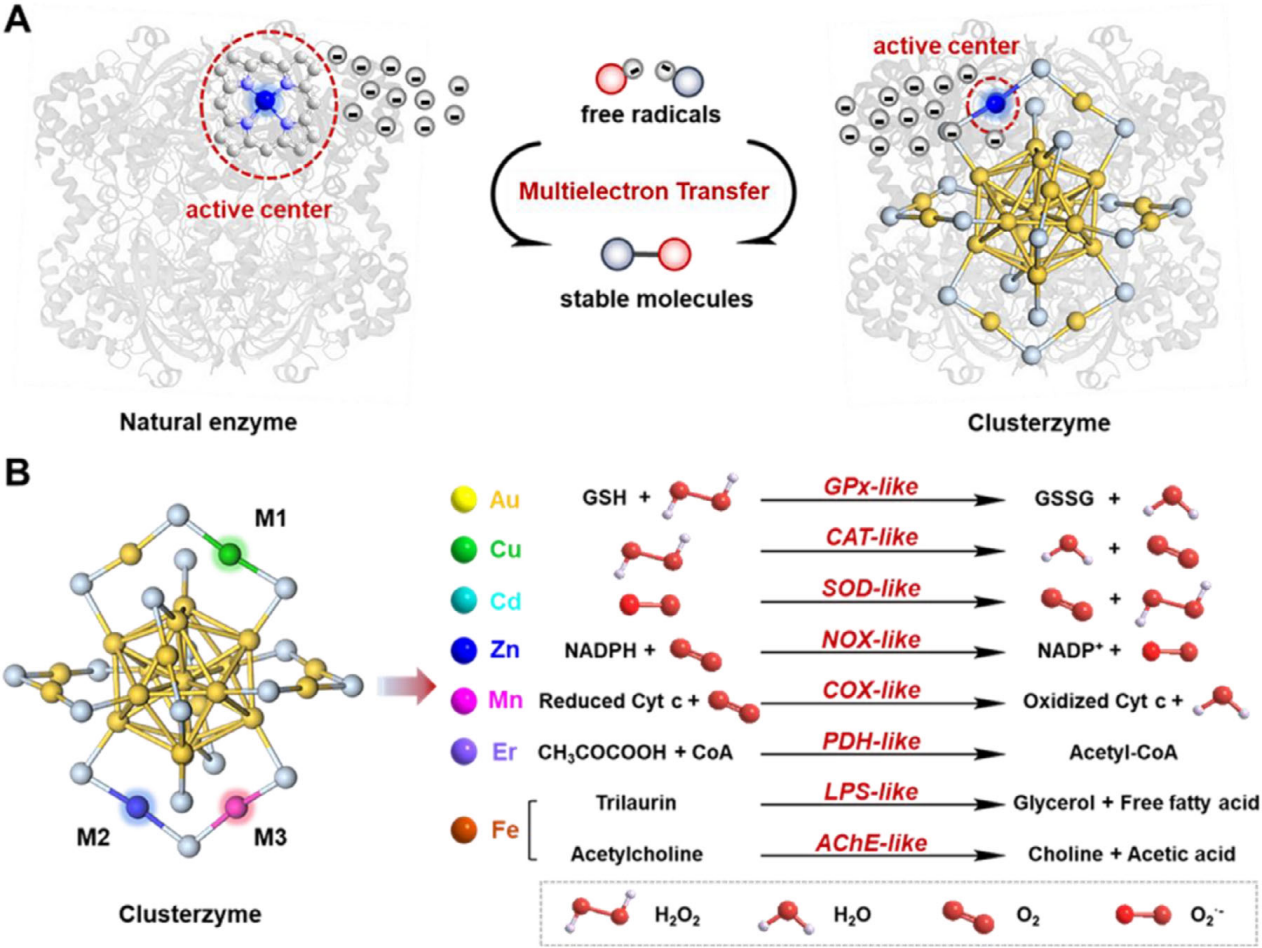
Concept, properties, programmability foundation of clusterzyme. A) Clusterzyme engineered through bioinspired active site design to facilitate multielectron transfer processes and scavenge free radicals. B) Enzymatic activity of Au_25_ series clusterzymes programmed via single‐atom doping strategies to achieve specific catalysis.

Based on this, the concept of the “clusterzyme” was formally introduced by Zhang's group in 2021, which refers to a programmable artificial enzyme constructed from an atomically precise metal cluster.^[^ ^47^ ^]^ Compared with traditional metal clusters, clusterzymes possess well‐defined atomic compositions, such as Au_25_(SR)_18_, Ag_44_(SR)_30_, crystallographic structures, and uniform sizes typically below 3 nm. Their atomic‐level structural precision endows them with tunable electronic properties, specific active sites, and predictable catalytic behaviors, which form the core basis for their design as biomimetic enzymes.^[^ ^40^, ^41^, ^42^ ^]^ The most potent designs often draw direct inspiration from biology, mimicking the multielectron transfer pathways and active‐site geometries of metalloenzymes. As illustrated in **Figure** **1**A, this bioinspired engineering enables clusterzymes to efficiently catalyze the conversion of reactive free radicals into benign molecules. Their emergence offers a highly designable and efficient platform for biomedical applications, including antioxidant therapy, neuroinflammation, and neuromodulation.^[^ ^48^, ^49^, ^50^, ^51^ ^]^

The transformative potential of clusterzymes lies in their programmability. Diverse enzymatic activities can be integrated through rational manipulation of the atomic architecture, primarily via atomic engineering and ligand engineering. Au_25_ clusters serve as a representative model, whose catalytic behavior can be tailored via single‐atom doping (Figure 1B). While Au_25_ exhibits glutathione peroxidase‐like (GPx‐like) activity, single‐atom substituted Au_24_Cu_1_ and Au_24_Cd_1_ show catalase‐like (CAT‐like) and superoxide dismutase‐like (SOD‐like) properties, respectively, enabling effective treatment of acute brain injury.^[^ ^47^ ^]^ This atomic‐level precision transcends mere mimicry, and it establishes a programmable platform where catalytic functions can be rationally encoded into the cluster structure to address specific biochemical challenges in complex diseases.

This review will demonstrate that clusterzymes represent more than just an improvement over previous artificial enzymes; they embody a shift from passive biocatalytic materials to actively programmable platforms. We first summarized the synthesis and characterization technologies of metal clusters, subsequently analyzed the atomic engineering and ligand engineering strategies to program their biocatalytic or enzyme‐like activity for neuroinflammation. Meanwhile, infrared emissive metal clusters were programmed with tunable electronic structure and optical properties at the atomic level for the early detection of diseases and clinical 3D visualization. Furthermore, semiconductor gold clusters were programmed with a rich electron carrier for a highly efficient brain–computer interface. Finally, we discuss challenges for the rational design and translational development of this programmable platform.

## 2 Synthesis and Characterization of Clusters

Atomically precise metal clusters with biocatalytic activities demand more challenging requirements in synthesis and characterization. In this section, the specific synthesis and characterization will be distinguished from those of other normal metal clusters, aiming to help understand the structure–activity relationship.

### 2.1 Synthesis

In the synthetic strategy of metal clusters, the wet chemical reduction is the most classical method. In general, under the presence of ligands, mild reductants, such as NaBH_4_ and CO are used to reduce the metal salt to metal clusters. For example, Au_25_(MPA)_18_ was prepared using CO at room temperature in an aqueous solution. Besides, Ag‐based (Ag_29_) and Cu‐based (Cu_30_) clusters that were synthesized through NaBH_4_ have been reported.^[^ ^52^, ^53^ ^]^ However, the clusters of this method have different sizes, which leads to poor yield. Etching has been proven to be an effective strategy in increasing the yield of clusters through improving selectivity and purity. It is achieved by adding an excess of ligands (such as GSH or PhC_2_H) after the reduction process is completed to allow the large‐sized clusters to gradually dissolve, rearrange, and eventually transform into the thermodynamically most stable magic number structure. In a typical procedure of Au_25_(SR)_18_, the yield of Au_25_ clusters was improved to 47%.^[^ ^54^ ^]^ Except for these, templates and assembly are common methods to prepare metal clusters. The multiple synthesis strategies provide a tailored foundation for atomic metal clusters with catalytic activities. However, these methods often face inherent challenges in scalability. The need for precise kinetic control, specific ligand‐to‐metal ratios, and multistep processes like etching currently constrains the throughput and reproducibility of gram‐scale synthesis.

Single‐atom substitution and ligand engineering effectively realize the catalytic activities of metal clusters as universal synthetic techniques. For instance, during the procedure of clusters, a heterometal salt was imported to the reaction system at the beginning. It ultimately formed Au_24_M_1_ clusters, which have different catalytic activities along with varying metals. In addition to heterometal substitution, ligand engineering offers a powerful avenue to tailor the catalytic functions of metal clusters. Mo et al. reported that Au_25_(NAC)_14‐17_(MPA)_4‐1_ clusters exhibited enhanced CAT‑like activity compared to the Au_25_(MPA)_18_ by replacing the original thiolate ligands with a hybrid shell of N‑acetyl‑L‑cysteine (NAC) and 3‑mercaptopropionic acid (MPA).^[^ ^55^ ^]^ More broadly, manipulating ligand size, donor strength, and binding mode has been shown to influence metal–ligand coordination, the interactions between ligands and surface accessibility, thereby modulating structural flexibility, charge transfer pathways, and catalytic site exposure in metal nanoclusters.^[^ ^56^ ^]^ In consequence, these synthetic strategies highlight that precise control over both the metal core and ligand shell is crucial for designing metal clusters with predictable and enhanced catalytic performance. Achieving such atomic‐level precision programmability, however, comes at the cost of synthetic complexity, presenting a significant challenge for large‐scale, cost‐effective production required for clinical translation.

### 2.2 Characterization

The characterizations of normal metal clusters, such as mass spectrum (MS), X‐ray photoelectron spectroscopy (XPS), and transmission electron microscope (TEM), etc., aim to confirm the composition, structure, and ligand environment. However, the dynamic monitor is otherwise needed in investigating the catalytic procedure of metal clusters. Specifically, the active sites, charge distribution, electric density, and the reaction intermediate are essential to reveal the catalytic pathway. Extended X‐ray absorption fine structure (EXAFS) and X‐ray absorption near‐edge structure (XANES) are widely used to study the local atomic structure, chemical state, and electronic structure information in clusters. The coordination of Au(I)‐S and Au(0)‐Au in Au_25_(SR)_18_ was revealed by EXAFS and XANES.^[^ ^57^ ^]^ In the last decades, density functional theory (DFT), as an effective technology, has gradually dominated in characterizing the precise structures of clusters. For example, the active sites and catalytic pathway of clusters have been defined by combining DFT and other characterization methods.^[^ ^58^, ^59^ ^]^ Generally, the catalytic mechanism of metal clusters involves electron transfer. Therefore, it is significant to capture the intermediate state during catalysis. Fourier transform infrared spectroscopy, Raman, and electron spin resonance are usual technologies in tracking intermediates.^[^ ^60^ ^]^ The comprehensive characterization combines the structures, activity, and mechanism of metal clusters. It enables the precise identification of active sites and guides the rational design of next‐generation clusters with enhanced efficiency and selectivity.

## 3 The Programmable Properties of Clusters

Atomically precise metal clusters offer programmable biocatalytic activities and tunable optical properties through rational design. This section details that atomic engineering and ligand engineering can facilitate this programmability, allowing for the systematic customization of cluster activities.

### 3.1 Engineering Oxidoreductase‐Like Ability

#### 3.1.1 Atomic Engineering

**Figure 2:**
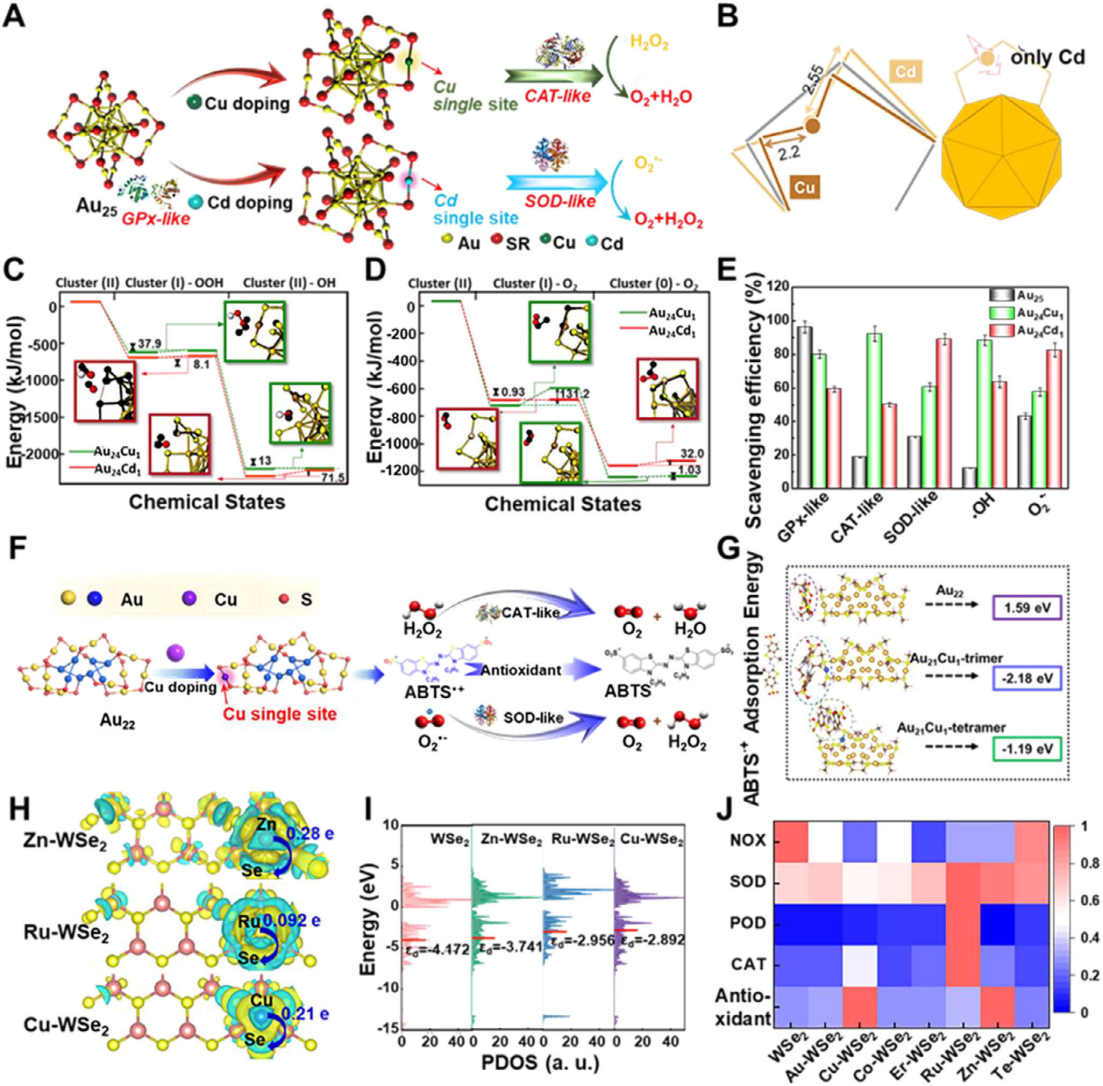
Programming oxidoreductase‐like activity in clusters through atomic engineering. A) Programming oxidoreductase‐like activity in Au_24_M_1_ clusters via single‐atom doping (M=Cu, Cd). B) A doped atom caused a change in bonds. C,D) Energy distribution diagrams and geometries of key intermediates and transition states for Au_24_Cu_1_ and Au_24_Cd_1_ in C) CAT and D) SOD simulated reactions. E) Corresponding quantifications of the scavenging efficiencies, confirming the functional specificity achieved by geometric programming. Reproduced under terms of the CC‐BY license.^[^ ^47^ ^]^ Copyright 2021, Haile Liu et al., published by Springer Nature. F) The activity regulation of Au_22_ clusters via single‐atom doping. G) Adsorption energy of Au_21_Cu_1_ cluster with ABTS^•+^. Reproduced with permission.^[^ ^61^ ^]^ Copyright 2023, The American Association for the Advancement of Science. H) Charge density difference for structure M‐WSe_2_ clusterzymes (M = Cu, Ru, Zn). I) TDOS and *d*‐band center of WSe_2_ and M‐WSe_2_ clusters. J) Heat map of the activity of WSe_2_ and M‐WSe_2_ clusters (M = Au, Cu, Co, Er, Ru, Zn, Te) for different catalytic reactions. Reproduced with permission.^[^ ^51^ ^]^ Copyright 2025, American Chemical Society.

Single‐atom doping constitutes a precise atomic engineering strategy for elucidating structure–activity relationships. By introducing distinct single atoms into Au_25_ clusters, our team achieved programmable enzyme‐like catalysis (**Figure** **2**A). Evidently, Au_25_, with distinct GPx‐like activity, demonstrated better performance in mimicking CAT and SOD separately after the substitution of Cu and Cd. Structurally, replacing the central Au atom with Cu shortens the Cu*─*S bond to 2.2 Å, creating a rigid active site that promotes H_2_O_2_ adsorption and decomposition, thereby enhancing CAT‐like activity. In contrast, Cd substitution elongates the bond to 2.55 Å, imparting structural flexibility that facilitates superoxide disproportionation and drives superior SOD‐like performance (Figure 2B). Thermodynamically, Au_24_Cu_1_ in the CAT pathway benefits from rigid Cu*─*S coordination, enabling low‐barrier H_2_O_2_ activation and high catalytic efficiency (Figure 2C), whereas Au_24_Cd_1_ in the SOD pathway leverages a flexible coordination environment to stabilize the transition state via conformational modulation, achieving pronounced SOD selectivity (Figure 2D). Quantitatively, the CAT‐like activity of Au_24_Cu_1_ increased by 80% compared to that of Au_25_. And the capacity of Au_25_ in mimicking SOD was improved to 90% after the Cd doping. Besides, Au_24_Cu_1_ and Au_24_Cd_1_ exhibit radical scavenging capacities 137‐ and 160‐fold higher than Trolox, respectively (Figure 2E).^[^ ^47^ ^]^ These atomic modifications not only govern reaction pathways and transition‐state stability but also translate into dramatic functional divergence. This geometric and electronic programming via single‐atom doping to enhance enzyme‐like activities is universal. In 2023, through DFT calculations, we confirmed that Cu single‐atom doping in Au_22_ clusters fundamentally enhanced the adsorption capacity for 2,2′‐Azinobis‐(3‐ethylbenzthiazoline‐6‐sulphonate) free radical (ABTS^•⁺^) by altering the surface electronic structure of the cluster (Figure 2G). Surprisingly, the Au_22_ cluster also performed SOD‐like and CAT‐like activities after Cu substitution (Figure 2F).^[^ ^61^ ^]^ Additionally, atomic engineering can precisely tune reactivity via electronic redistribution. The charge density of M‐WSe_2_ clusters in Figure 2H reveals that the introduction of Cu, Ru, and Zn elements alters the local bonding environment. Metal doping induces electron‐rich regions on the cluster surface, which are conducive to the occurrence of catalytic reactions. Furthermore, the total density of states (TDOS) analysis suggests that single‐atom doping results in an upward shift of the d‐band center (Figure 2I), thereby enhancing substrate binding and programming distinct catalytic selectivity. Cu‐WSe_2_ and Zn‐WSe_2_ favor antioxidant activity, Ru‐WSe_2_ and Cu‐WSe_2_ enhance CAT‐ and POD‐like activity, while pristine or Te‐WSe_2_ clusters prefer NADH oxidase‐like (NOX‐like) behavior (Figure 2J).^[^ ^51^ ^]^ Therefore, different metal substitution effectively regulates the variational enzymatic activities.

Besides, synergistic enzymatic activities can be achieved on a single cluster through atomic engineering. Fan et al. developed Au_1_Pd_3_ alloy nanozymes integrated with SOD‐ and myeloperoxidase (MPO)‐like activities, which exhibit excellent tumor therapeutic performance. In this case, the activity programmability is regulated by varying the ratio of Au and Pd is 1:3.^[^ ^62^ ^]^ Our recent work reported Ce_12_V_6_ clusters with multiple enzymatic activities, such as mimicking GPx, SOD, and POD activities. Attentionally, the multienzymatic activities were realized by incorporating the characteristics of Ce and V.^[^ ^63^ ^]^ These unequivocally highlight the power of atomic‐level structural tuning in programming enzyme‐mimetic catalysis.

#### 3.1.2 Ligand Engineering

**Figure 3:**
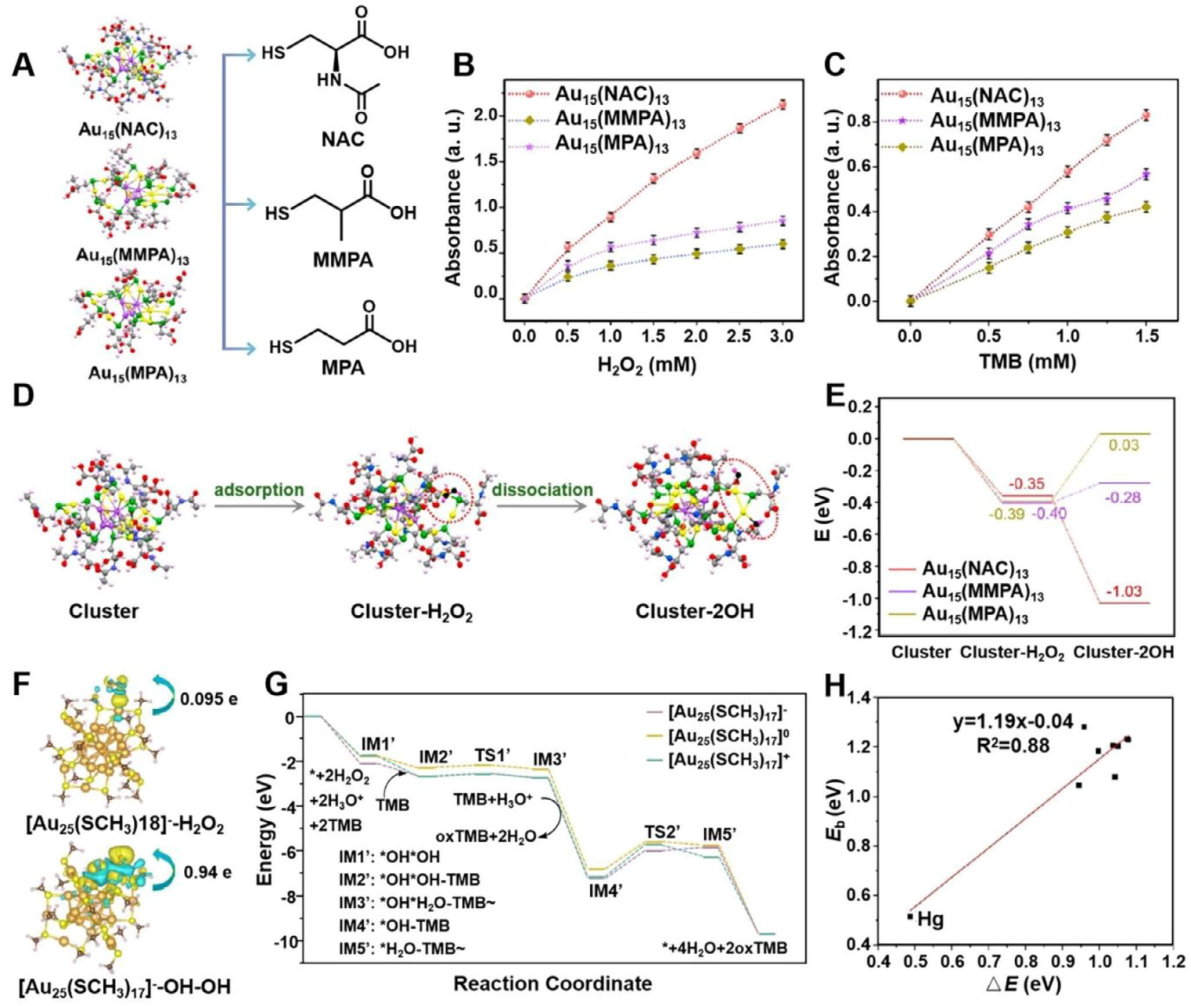
Programming oxidoreductase‐like activity in clusters through ligand engineering. A) Programming oxidoreductase‐like activity in Au_15_ clusters protected by different thiolate ligands (NAC, MMPA, MPA). B‐C) POD mimic catalytic activity of Au_15_(NAC)_13_, Au_15_(MMPA)_13_, Au_15_(MPA)_13_ at 652 nm against B) H_2_O_2_ and C) TMB. D) Schematic illustration of the proposed catalytic pathways of Au_15_(NAC)_13_ clusters. E) Energy profiles for the catalyzed dissociation of H_2_O_2_ by Au_15_(NAC)_13_, Au_15_(MMPA)_13_, Au_15_(MPA)_13_. Reproduced with permission.^[^ ^64^ ^]^ Copyright 2023, American Chemical Society. F) The charge difference density for H_2_O_2_ adsorption on [Au_25_(SCH_3_)_18_]^−^ and adsorption of dissociated *OH−*OH for [Au_25_(SCH_3_)_17_]^−^. G) The relative energy profile of the POD‐like catalytic reaction on [Au_25_(SCH_3_)_17_]^q^. H) The Bronsted–Evans–Polanyi relationship between Δ*E* and *E*_b_ of the rate‐determining step on the partially dethiolated [MAu_24_(SCH_3_)_17_]^q^ nanoclusters along the POD pathway. Reproduced with permission.^[^ ^65^ ^]^ Copyright 2025, American Chemical Society.

Beyond atomic doping, ligand engineering serves as a powerful strategy to program cluster activity by modulating the catalytic microenvironment. This approach precisely controls substrate access, intermediate stabilization, and electron transfer, thereby influencing both reaction efficiency and pathway selectivity. Xie et al. verified that the ligand on the clusters could significantly affect their POD‐like activity (**Figure** **3**A). Among the three different thiol ligands NAC, 3‐mercaptopropionic acid (MPA), or 3‐mercapto‐2‐methylpropanoic acid (MMPA), Au_15_(NAC)_13_ cluster exhibits optimal biocatalytic efficiency toward both substrates H_2_O_2_ and TMB (Figure 3B,C), representing the optimal POD‐like activity, and detailed enzymatic kinetics data further show that its catalytic activity is about 4.3 and 2.7 times higher than that of Au_15_(MMPA)_13_ and Au_15_(MPA)_13_, respectively. By use of DFT, the complete biocatalytic pathway for H_2_O_2_ adsorption‐dissociation during POD‐mimetic catalysis is depicted in Figure 3D. Additionally, H_2_O_2_ dissociation constitutes the rate‐determining step in this biocatalytic process. Au_15_(NAC)_13_ cluster minimizes the dissociation energy barrier of H_2_O_2_, which is the core mechanism underlying the differential POD‐mimetic activity of Au_15_(NAC)_13_.^[^ ^64^ ^]^ Recently, DFT has been considered as an effective method to investigate the structure–activity relationship. In 2025, Tang et al. systematically investigated the regulatory mechanisms governing POD in the gold nanocluster Au_25_(SCH_3_)_18_ under conditions of ligand removal through first‐principles calculations. Changes in the number of ligands induce alterations in the charge difference density around the clusters (Figure 3F). Removal of the ligand moiety reconfigures the catalytic pathway and reduces the overall energy barrier (Figure 3G). For anionic [Au_25_(SCH_3_)_17_]^−^, this shifts the POD catalysis rate‐determining step and lowers its barrier to 1.20 eV via exposed undercoordinated Au sites, while boosting H_2_O_2_ dissociation. This validates that ligand regulation constitutes an effective strategy for enhancing catalytic performance (Figure 3H).^[^ ^65^ ^]^ This concept is reinforced by broader work. Fang et al. confirmed that ligands can alter the electronic structure of surface atoms, tune adsorption energies of intermediates, and selectively expose or block active sites, thereby dictating reaction kinetics and product distribution.^[^ ^66^ ^]^ Similarly, Wang et al. demonstrate that subtle variations in ligand chemistry or steric configuration can shift the rate‐determining step in redox reactions and enhance turnover frequency by orders of magnitude, which is consistent with the ligand‐modulation effects of atomically precise [Au_25_(SR)_18_]^−^. Specifically, hydrophilic ligands (e.g., −SCH_2_COOH) improve acidic HER performance, while hydrophobic ligands (e.g., −SCH_2_CH_3_) optimize alkaline CO_2_ reduction reaction (CO_2_RR) efficiency, highlighting the core regulatory role of ligands in the redox catalysis of nanoclusters.^[^ ^67^ ^]^ These examples demonstrate that ligands can fine‐tune the electronic structure of active sites, modulate intermediate adsorption energies, and impose steric control over substrate access. Furthermore, the ligand can play a vital role in modulating activities as an active site. For example, Zhang et al. designed a Ni‐3,3′‐diaminobenzidine ligand (Ni‐DAB) that mimicked the dual‐site catalytic mechanism of urate oxidase (UOX). In this work, the β‐C in the DAB ligands acted as an active site to specifically catalyze uric acid.^[^ ^68^ ^]^ It affords a novel track for programming activities of clusters. These results confirmed that appropriately setting the parameters of atomic engineering and ligand engineering, specific enzyme‐mimicking activities can be activated, which provides tremendous potential for designing clusters with customized biocatalytic properties.

### 3.2 Engineering Nonoxidoreductase‐Like Activity

The programmability of clusters extends their functional repertoire beyond redox chemistry into the realm of nonredox catalysis, including hydrolysis and group transfer reactions.^[^ ^69^, ^70^ ^]^ This expansion is achieved by programming Lewis acidic metal active centers and designing cooperative ligand environments that work in concert to activate substrates and stabilize transition states.^[^ ^71^, ^72^ ^]^

**Figure 4:**
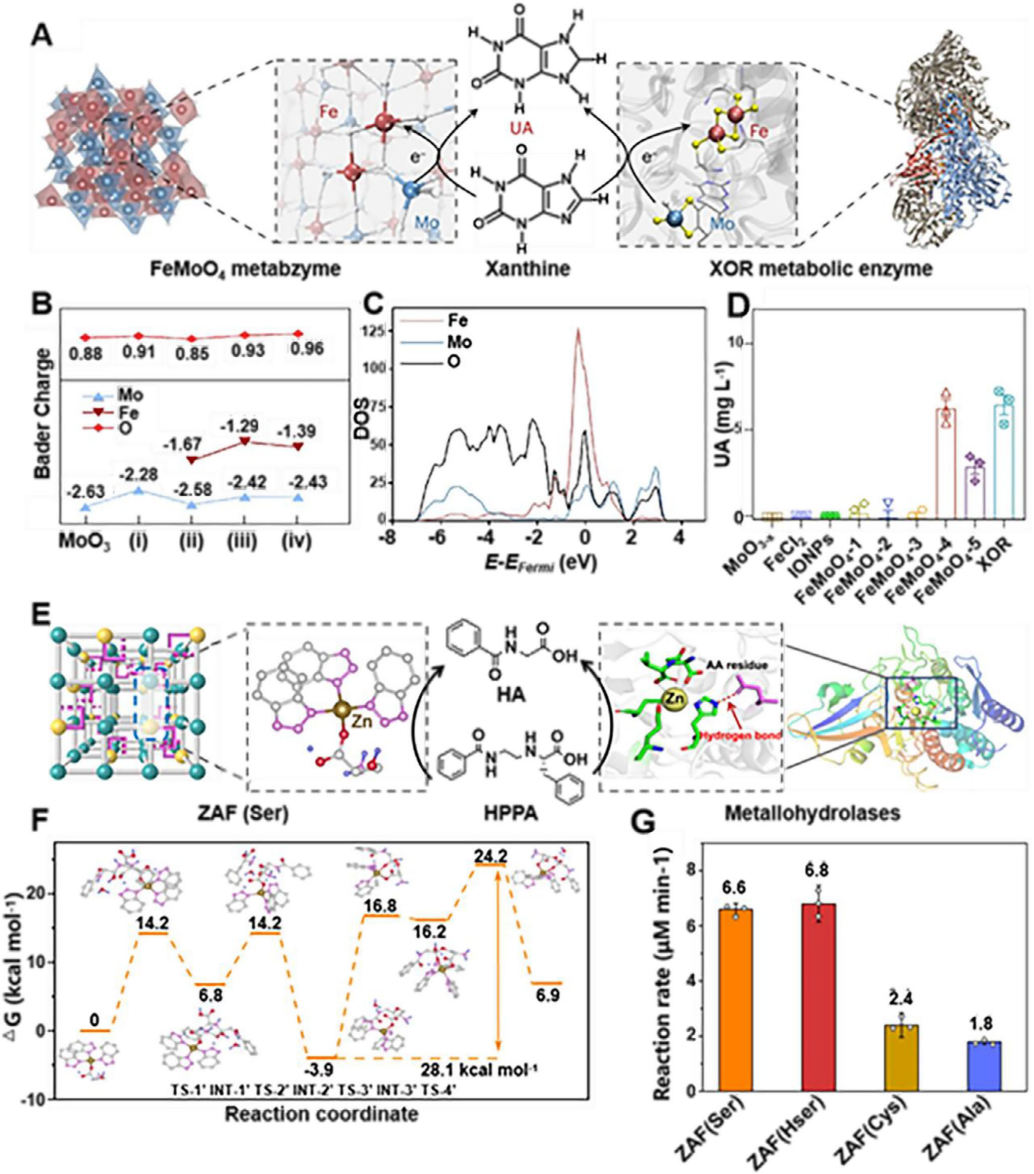
Programming nonoxidoreductase‐like activity in metabzyme. A) Design diagram of the FeMoO_4_ metabzyme mimicking xanthine oxidoreductase (XOR). B) Bader charge variations of O, Mo, Fe atoms in multimetal metabzyme during the atomic and crystal structure rearrangement process. C) DOS. D) Generation of uric acid (UA) demonstrating the high activity of the programmed FeMoO_4_ metabzyme compared to controls. Reproduced with permission.^[^ ^73^ ^]^ Copyright 2024, Springer Nature. E) Design diagram of the serine‐incorporated Zn azolate framework (ZAF(Ser)). F) Gibbs free energy curve showing the reduced barrier via the hydrogen bond‐mediated catalytic pathway. G) Catalytic activities of ZAF(Ser, ZAF(Hser, ZAF(Ala, ZAF(Cys, showcasing the enhanced activity of the programmed ZAF(Ser). Adapted under terms of the CC‐BY license.^[^ ^78^ ^]^ Copyright 2023, Xin Yuan et al., published by Springer Nature.

Programming multimetal centers enables the mimicry of complex metabolic enzymes. A seminal example is that Ling et al. reported the Fe‐doped MoO_4_ that mimics xanthine oxidoreductase (XOR).^[^ ^73^ ^]^ This system is programmed with synergistic Fe^2+^ and tetrahedral Mo^4+^ active sites. During its formation, Mo vacancies are created to adsorb Fe atoms, leading to asymmetric oxygen release and the final FeMoO_4_ structure, with the active center validated by Bader charge analysis (**Figure** **4**A,B). In this programmed cycle, the Fe site facilitates electron transfer, while the Mo center undergoes a reversible Mo^4+^/Mo^5+^ redox cycle to cleave the C*─*H bond of xanthine, driving its conversion to uric acid (Figure 4C). The programmed bimetallic center exhibits significantly higher activity than monometallic analogues, highlighting the power of rational element selection (Figure 4D).^[^ ^73^, ^74^, ^75^, ^76^ ^]^ Beyond redox‐metal partnerships, programming strong Lewis acid centers provides a versatile route to hydrolytic catalysis. Wei et al. constructed a Ce‐based cluster that exhibits broad‐spectrum hydrolytic activity. It efficiently cleaves phosphate ester bonds in nucleotides, amide bonds in peptides, and glycosidic bonds in polysaccharides. The Ce ions provide Lewis acidic sites that activate substrates toward nucleophilic attack, while surrounding ligands modulate substrate access and product release. These clusters show particular promise in therapeutic applications requiring degradation of pathological aggregates.^[^ ^77^ ^]^

Notably, ligands act as crucial regulatory units for metal cluster structure and function, modulating the electronic structure of the cluster, influencing its geometric structure through steric hindrance and coordination patterns, and playing a pivotal role in optimizing catalytic performance. Yuan et al. developed the serine‐incorporated Zn azolate framework (ZAF(Ser)), which is a masterful demonstration of this principle (Figure 4E).^[^ ^78^ ^]^ Its programmability lies in the partnership between benzotriazole (BTA) and serine ligands. The BTA enhances the Lewis acidity of Zn^2+^, while the serine provides a hydrogen‐bonding network that activates a hydroxyl nucleophile. This programmed cooperation reduces the amide hydrolysis energy barrier from 42.0 to 28.1 kcal mol^−1^ (Figure 4F), resulting in a 3.2‐fold activity enhancement over the ligand‐free ZAF, showcasing how ligand environments can be engineered to create powerful artificial hydrolases (Figure 4G).

Emerging applications further demonstrate the functional diversity that can be achieved through programming. Chen et al. emulated enzymes like carbonic anhydrase (CA) through programmed Zn_3_ active sites that position hydroxyl nucleophiles for CO_2_ hydrolysis. The peripheral Zn_3_ in the cluster provides a coordination environment similar to the active site of CA, enabling nucleophilic attack on substrates by activating hydroxyl groups (OH^−^). Meanwhile, cluster size reduction enhances substrate contact efficiency. These two strategies work in conjunction to jointly achieve a significant improvement in catalytic performance.^[^ ^74^, ^75^, ^76^ ^]^ Similarly, Chen et al. developed a cluster that mimics catechol oxidase through dinuclear Cu‐(OH)‐Cu motifs programmed for aerobic oxidation via a deprotonation mechanism, efficiently bypassing H_2_O_2_ production.^[^ ^78^, ^79^ ^]^ In summary, the successful mimicry of nonoxidoreductases solidifies the status of clusters as a fully programmable catalytic platform, capable of addressing a wide spectrum of biochemical challenges through atomic‐level design.

### 3.3 Tunable Optical Properties of Metal Clusters at the Atomic Level

The programmability of clusters culminates in the rational design of their photoluminescent properties, enabling their evolution into the detection of diseases. In recent years, Au_25_(SR)_18_, Au_42_, Au_52_ clusters, and so on, have been reported to exhibit luminescent properties in the second near‐infrared window (NIR‐II).^[^ ^80^, ^81^, ^82^, ^83^, ^84^, ^85^, ^86^ ^]^ Tunable optical properties can also be engineered by atomic engineering and ligand engineering.

**Figure 5:**
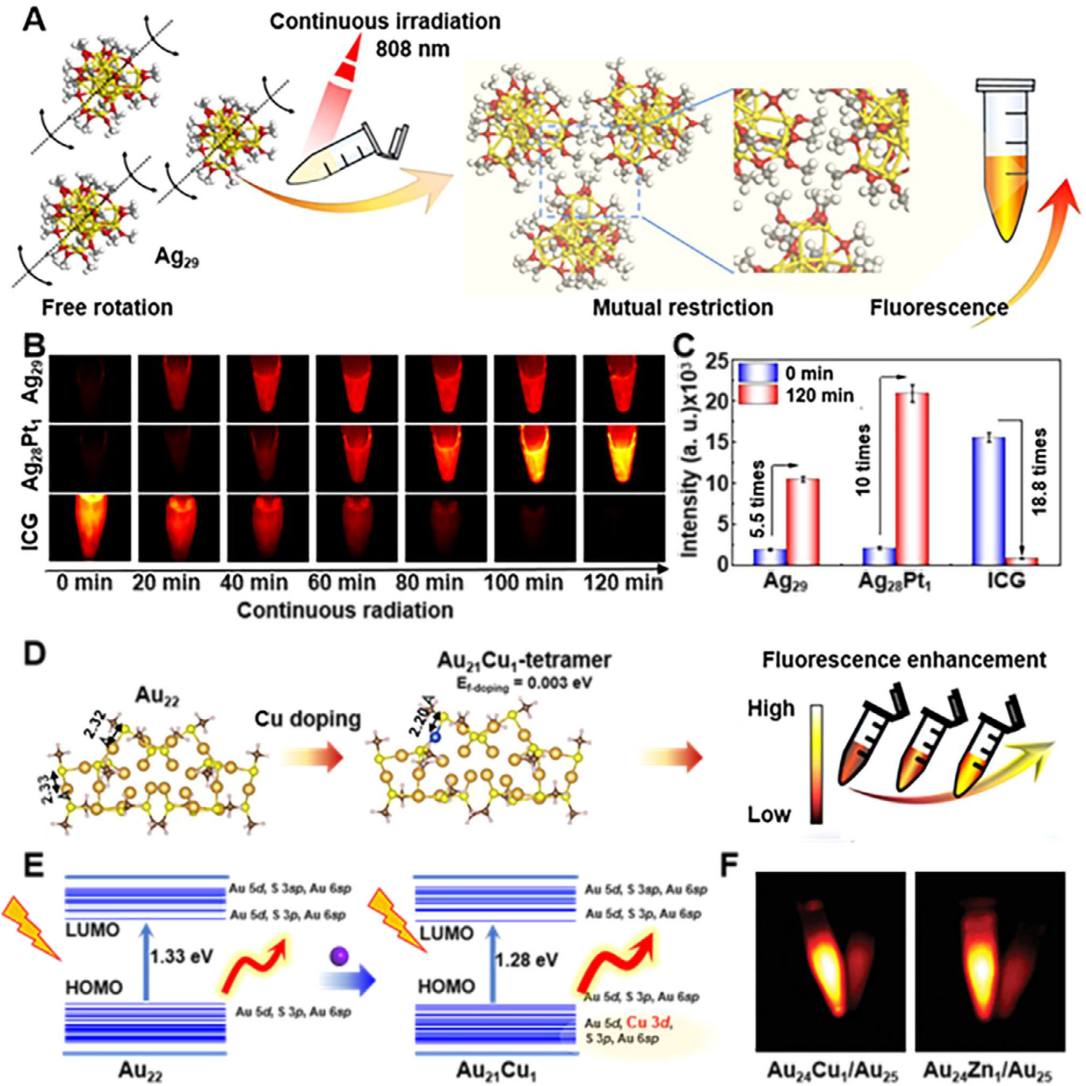
Tunable optical properties of metal clusters. A) The Ag_29_ cluster exhibits aggregation‐enhanced fluorescence. B,C) Fluorescence images of the Ag_29_, Ag_28_Pt_1_, ICG under continuous radiation by 808 nm laser (excitation: 808 nm; filter: 1000 nm long pass; exposure time: 100 ms). Reproduced with permission.^[^ ^53^ ^]^ Copyright 2024, Elsevier. D) Cluster luminescence regulation based on atomic doping. E) The energy level diagram of Au_22_ and Au_21_Cu_1_ clusters. Reproduced with permission.^[^ ^61^ ^]^ Copyright 2023, The American Association for the Advancement of Science. F) NIR‐II fluorescence image of an aqueous solution of Cu and Zn doped Au_25_ clusters. Reproduced with permission.^[^ ^87^ ^]^ Copyright 2019, John Wiley and Sons.

Precise control over the elemental composition of clusters enables direct programming of their electronic structure, thereby promoting radiative relaxation and consequently influencing their optical properties. For instance, doping the single atom Pt into Ag_29_ cluster to form Ag_28_Pt_1_ does not merely introduce 5*d* orbitals, but also hybridizes these orbitals with the original electronic structure, leading to a wider highest occupied molecular orbital (HOMO)‐lowest unoccupied molecular orbital (LUMO) gap and the creation of distinct, lower‐energy excited states. This atomic‐level programming significantly enhances luminescence efficiency and photostability, resulting in a tenfold fluorescence enhancement over 120 min under 808 nm excitation (**Figure** **5**A–C).^[^ ^53^ ^]^ The same principles apply to Au clusters. Cu doping in Au_21_Cu_1_ shortened S‐Au‐S chains into bent S‐Cu‐S motifs, introduced 3*d* orbitals near the HOMO, and maintained high stability (0.003 eV), simultaneously optimizing NIR‐II emission and enzyme‐mimetic activity for in vivo imaging (Figure 5D–F).^[^ ^61^ ^]^ These examples underscore atomic engineering as a generalizable strategy for creating bright, stable, and theranostical active emitters.

Ligand shell offers a complementary and powerful route to program optical characteristics by primarily influencing the nonradiative relaxation pathways. The key mechanism is the rigidification of the structure and the modulation of surface states of clusters. In Au_25_(Cys)_18_, the optimal electron donation from cysteine ligands strengthens the Au*─*S bond, which restricts the vibrational and rotational freedom of the ligand‐shell interface. This rigidity, in turn, reduces the rate of nonradiative vibrational energy loss, thereby enhancing radiative recombination.^[^ ^87^ ^]^ Building on this principle of suppressing molecular motion through rigid coordination environments, Dai et al. conjugated Au clusters with zwitterionic phosphorylcholine ligands, improving solubility and biocompatibility while stabilizing surface states, thereby boosting NIR‐II emission for lymph node imaging in cancer models.^[^ ^88^ ^]^ Yang et al. developed protein‐biomodified Au clusters, where rigid coordination environments suppressed vibrational relaxation and promoted radiative recombination, enabling bright tumor‐targeted imaging.^[^ ^89^ ^]^ Beyond biomolecular engineering, Wang et al. achieved near‐unity NIR phosphorescence in solvated Au_16_Cu_6_ clusters by ligand‐mediated orbital hybridization and rigidification, which efficiently suppressed nonradiative pathways.^[^ ^90^ ^]^

In conclusion, the precise engineering of both the cluster core and the ligand shell enables the creation of bright, stable, and biocompatible optical probes. This precise control over optical properties at the atomic level establishes clusters as a unique platform for advanced biomedical imaging and diagnostics, particularly in neuroscience, where high‐resolution visualization of complex neural structures and dynamic processes is essential. Some typical atomic precise clusters and their characteristics are listed in **Table** **1**.

**Table 1:** Typical atomic precise clusters and their core properties and references.

Clusters	Core properties	Refs.
Au_25_(MPA)_18_	Antioxidant activity, SOD	[47]
Au_24_Cd_1_(MPA)_18_	Antioxidant activity, GPx	[47]
Au_24_Cu_1_(MPA)_18_	Antioxidant activity, CAT	[47]
Au_24_Ag_1_(MPA)_18_	Antioxidant activity, CAT	[100]
Au_15_(NAC)_13_	POD	[64]
Au_25_(SCH3)_18_	POD	[65]
Cu‐WSe_2_	Antioxidant activity, POD	[51]
Zn‐WSe_2_	Antioxidant activity	[51]
Ru‐WSe_2_	CAT	[51]
Te‐WSe_2_	NOX	[51]
FeMoO_4_	XOR	[73]
Ce (fumaric acid)	Hydrolytic	[77]
ZAF(Ser)	Hydrolytic	[78]
Zn_3_	CA	[74]
Cu_2_O_2_	Catechol oxidase	[79]
Au_25_(SG)_18_	NIR‐II photoluminescence	[87]
Au_25_(Cys)_18_	NIR‐II photoluminescence	[87]
Au_21_Cu_1_(SG)_16_	NIR‐II photoluminescence	[87]
Au_24_Pr_1_	NIR‐II photoluminescence	[128]
Ag_28_Pt_1_	NIR‐II photoluminescence	[53]
Au_44_	NIR‐II photoluminescence	[129]
Au_52_(SR)_32_	NIR‐II photoluminescence	[86]
Au(phosphorylcholine)	NIR‐II photoluminescence	[88]
Au(protein)	NIR‐II photoluminescence	[89]
Au_16_Cu_6_	NIR phosphorescence	[46]

## 4 Metal Clusters in Neuroscience

The programmability of clusters, achieved through precise atomic and ligand engineering, endows them with tailored biocatalytic, optical, and electronic properties that are uniquely suited to address complex challenges in neuroscience.^[^ ^46^, ^91^ ^]^ By enabling targeted modulation of neuroinflammatory pathways, high‐resolution deep‐tissue imaging, and enhanced neural interface performance, these atomically defined clusters offer a versatile platform for both understanding and intervening in neurological disorders. This section explores how such programmable functionalities are harnessed for therapeutic, diagnostic, and bioelectronic applications in the central nervous system, highlighting their transformative potential in advancing neuroscience research and clinical practice.

### 4.1 Clusters for Treatment of Central Nervous Diseases

The treatment of central nervous diseases demands interventions capable of precisely modulating the complex pathological microenvironment, mainly defined by oxidative stress and neuroinflammation.^[^ ^92^, ^93^, ^94^ ^]^ Clusters directly address this challenge through their programmable biocatalytic activities and favorable biosafety, which can be rationally selected to target specific, dysfunctional pathways with high precision.

#### 4.1.1 Neuroinflammation

Neuroinflammation is a critical pathological driver in numerous central nervous system disorders, characterized by the excessive activation of glial cells and elevated levels of proinflammatory cytokines. These processes are often initiated and exacerbated by a burst of diverse reactive oxygen species (ROS), thereby in turn triggering severe inflammation, leading to neuronal damage and synaptic dysfunction.^[^ ^95^, ^96^, ^97^, ^98^, ^99^ ^]^ This complexity renders broad‐spectrum antioxidants suboptimal, as they lack the precision to selectively interrupt specific damaging cascades without disrupting essential redox signaling.

**Figure 6:**
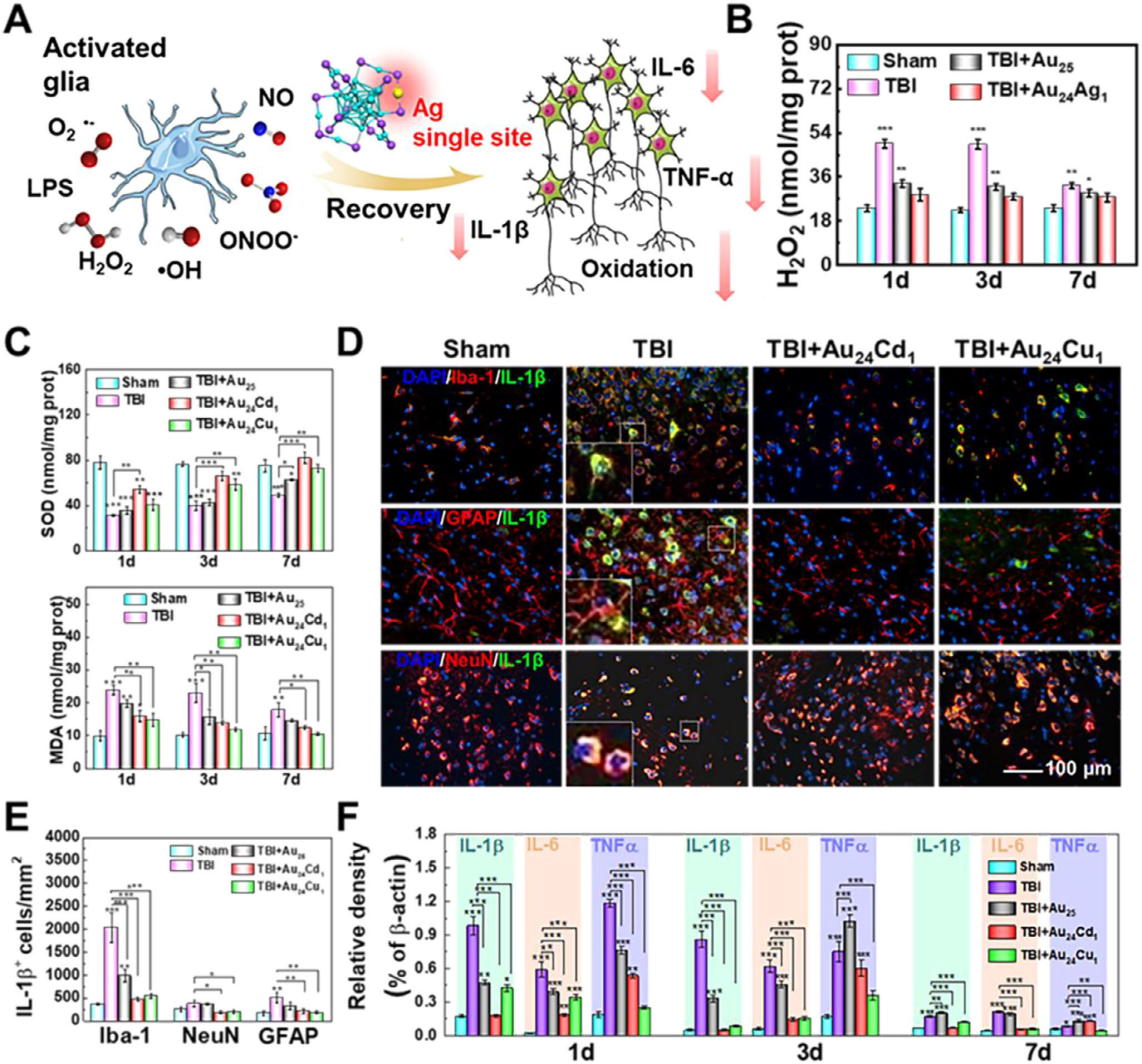
Clusters programmed for neuroinflammation. A) Schematic illustration of oxidative stress and inflammatory responses modulated by clusterzymes after stimulation. B) Improvement of oxidative stress indicators by Au_24_Ag_1_ clusterzymes at different time points postinjury. Reproduced with permission.^[^ ^100^ ^]^ Copyright 2021, American Chemical Society. C) Improvement of oxidative stress indicators. D) Colocalization of IL‐1β with microglia (Iba‐1, astrocytes (GFAP, or neurons (NeuN) and E) quantitative analysis showing Au_24_Cd_1_ primarily targets IL‐1β expression in microglia and neurons. F) Quantitative analysis of western blotting. Reproduced with permission.^[^ ^47^ ^]^ Copyright 2021, Haile Liu et al., published by Springer Nature.

To address this need for precision, the cluster platform was strategically programmed for catalytic specificity. Our group harnessed the principle that single‐atom doping can precisely tune the geometric and electronic structure of the catalytic core. Our group has developed Au_24_Ag_1_ clusters through Ag substitution, which exhibited enhanced radical scavenging capacity, illustrating the preliminary step toward activity tuning (**Figure** **6**A,B).^[^ ^100^ ^]^ Meanwhile, our group has yielded a suite of gold clusters with distinct enzymatic preferences through the rational design that Au_24_Cd_1_ was engineered for superior SOD‐like activity by facilitating O_2_^•−^ disproportionation, while Au_24_Cu_1_ was optimized for potent CAT‐like activity that favors the stable adsorption and cleavage of H_2_O_2_ (Figure 6C).^[^ ^47^ ^]^ This precise functional programming yielded distinct and insightful biological outcomes. As shown in Figure 6D–F, the SOD‐programmed Au_24_Cd_1_ preferentially targeted superoxide and nitrogen‐containing signaling molecules, leading to a significant suppression of interleukin (IL)‐6and IL‐1β expression. In contrast, the CAT‐programmed Au_24_Cu_1_ primarily scavenged peroxides, resulting in a marked reduction in tumor necrosis factor (TNF)‐α levels.^[^ ^47^ ^]^ These distinct results directly demonstrate that programmable clusters are powerful tools. They deliver precise treatment while also helping us untangle the complex inflammatory networks caused by ROS. Additionally, Xiao et al. further demonstrated that surface functionalization with dihydrolipoic acid (DHLA‐AuNCs) can program an anti‐inflammatory phenotype. This effect was achieved by inducing M2‐like microglial polarization through downregulation of NF‐κB signaling, showcasing how ligand engineering can complement metal core doping to tailor biological responses.^[^ ^101^ ^]^ Stevens et al. engineered gold clusters with POD‐like activity conjugated to a voltage‐gated Na^+^ channel (NAv) protein scaffold via surface functionalization, which can rapidly respond to the inflammatory conditions and achieve the detection.^[^ ^102^ ^]^ This clear dissociation of effects demonstrates the power of clusters as platforms to not only achieve therapeutic efficacy but also to deconvolute complex pathological processes by acting as a selective molecular tool.

#### 4.1.2 Neurodegenerative Diseases

Alzheimer's disease (AD) and Parkinson's disease (PD) are prototypical neurodegenerative disorders whose intractability stems from their multifactorial nature, involving interconnected pathologies of protein misfolding, oxidative stress, neuroinflammation, and eventual neuronal loss.^[^ ^103^, ^104^, ^105^ ^]^ The limitation of current therapies lies in their single‐target approach, which is inadequate against, such a complex, self‐reinforcing pathological network.

**Figure 7:**
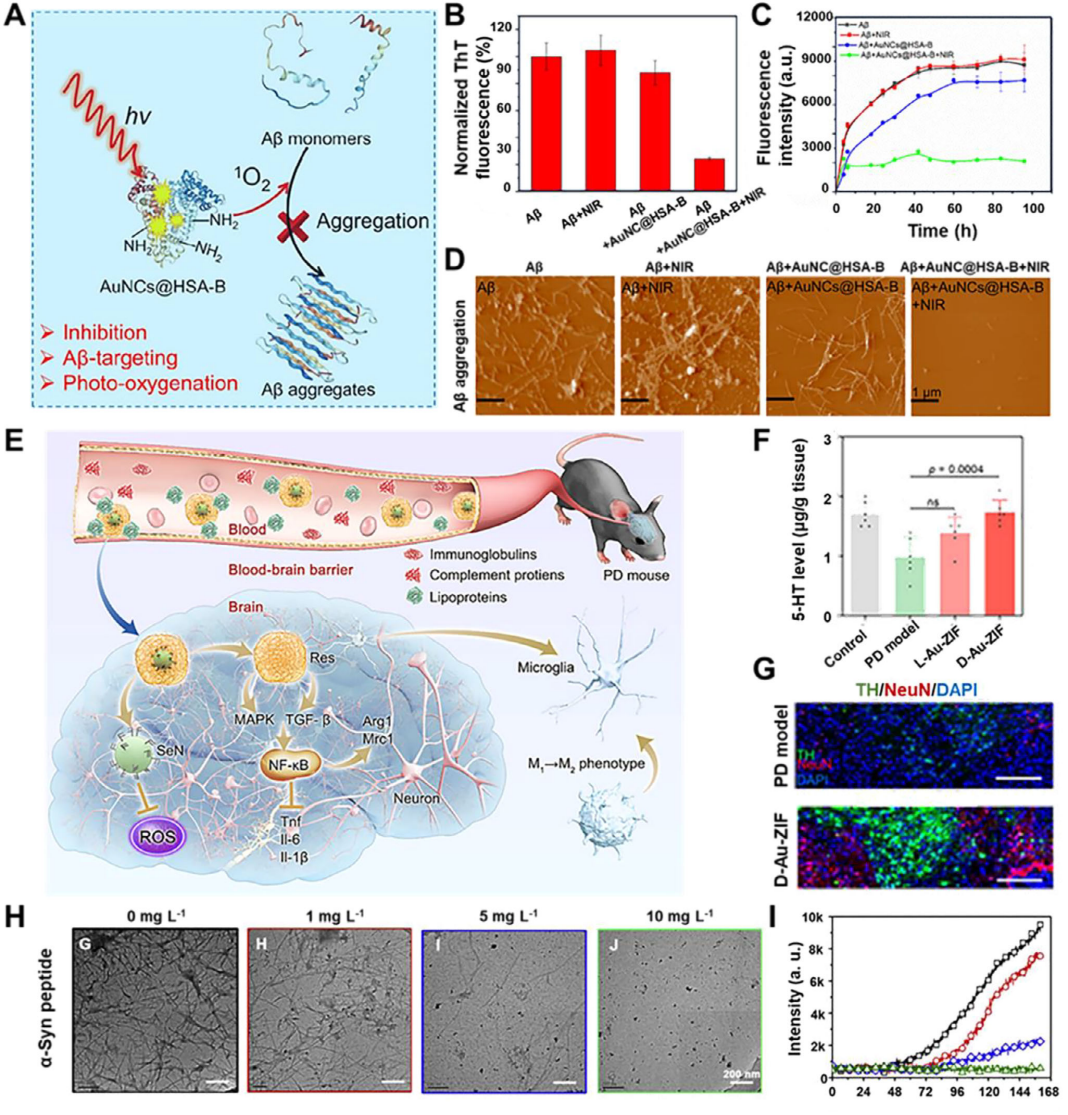
Clusters programmed for neurodegenerative diseases. A) Design of AuNCs@HSA‐B for inhibiting Aβ aggregation and scavenging ROS. B) Normalized final ThT fluorescence intensities. C) Time‐dependent ThT fluorescence changes. D) Atomic force microscope (AFM) images of Aβ after various interventions. Reproduced with permission.^[^ ^111^ ^]^ Copyright 2022, Elsevier. E) Schematic of PD pathology and intervention through antioxidant activity and metabolic reprogramming. Reproduced with permission.^[^ ^114^ ^]^ Copyright 2025, Elsevier. F,G) Clusterzyme‐loaded ZIF frameworks program striatal 5‐HT levels and tyrosine hydroxylase (TH) expression, promoting dopaminergic recovery. Reproduced under terms of the CC‐BY license.^[^ ^115^ ^]^ Copyright 2025, Junyang Chen et al., published by John Wiley and Sons. H,I) L‐NIBC‐protected clusterzymes programmed to directly inhibit α‐synuclein (α‐Syn) aggregation and fibrillation. Black: 0; Red: 1 mg L^−1^; Blue: 5 mg L^−1^; Green: 10 mg L^−1^. Reproduced with permission.^[^ ^116^ ^]^ Copyright 2018, Elsevier.

The cluster platform has been programmed to simultaneously engage several of these pathological nodes, offering a unified therapeutic strategy. In AD, the dual insult of amyloid‐beta (Aβ) plaque deposition and concomitant oxidative stress is a primary target.^[^ ^106^, ^107^, ^108^, ^109^, ^110^ ^]^ Sun et al. designed a sophisticated system where basified human serum albumin (HSA‐B) was used to stabilize gold clusterzymes (AuNc@HSA). This design programmed dual functionalities that the HSA‐B platform inherently inhibits Aβ fibrillization, while the gold cluster scavenges ROS, alleviating Aβ‐mediated neurotoxicity at low concentrations (**Figure** **7**A–D).^[^ ^111^ ^]^ Expanding on this, Tang et al. reported that chiral glutathione (GSH)‐stabilized gold clusters were shown to inhibit neuronal death and rescue memory impairments by suppressing Aβ42 aggregation and mitigating ROS.^[^ ^112^ ^]^ This ligand engineering facilitated the interaction of gold clusters with the KLVFFA segment of Aβ via hydrogen bonding, which is the widely accepted key element for forming Aβ aggregation.^[^ ^112^ ^]^ Taking a more direct approach against established pathology, Cys‐Arg (CR) dipeptide‐modified gold clusters (Au_23_(CR)_14_) were engineered to actively dissolve preformed endogenous Aβ plaques and restore the natural unfolded state of Aβ peptides, demonstrating the programmability for direct protein remodeling, a function beyond mere protection.^[^ ^113^ ^]^

Similarly, for PD, defined by the loss of dopaminergic neurons and the accumulation of α‐synuclein (α‐Syn), cluster designs have been programmed for multifaceted efficacy (Figure 7E).^[^ ^110^, ^114^ ^]^ Li et al. employed a combinatorial strategy by loading gold clusters into zeolitic imidazolate framework (ZIF) frameworks. This system was designed to reprogram microglial metabolism to induce an anti‐inflammatory response, while also boosting dopamine (DA) synthesis by remarkably increasing tyrosine hydroxylase (TH) levels (Figure 7F–I).^[^ ^115^ ^]^ In a more targeted design against the core pathology, *N*‐isobutyryl‐L‐cysteine (L‐NIBC)‐protected gold clusters were specifically engineered to prevent α‐Syn aggregation and fibrillation, which in turn reversed DA neuron loss and ameliorated behavioral deficits in PD mice.^[^ ^116^ ^]^ These examples underscore the exceptional versatility of the clusters. It can be precisely programmed to tackle the complex, multifactorial nature of neurodegenerative diseases, from inhibiting aggregation and counteracting oxidative stress to modulating neuroinflammation, all within a single, rationally designed nanoscale entity.

### 4.2 Clusters for the Detection of Central Nervous Diseases

The programmability of clusters extends beyond biocatalysis to the realm of bioimaging, where their tunable optical properties and surface chemistry can be engineered to create highly specific and informative contrast agents. This capability is particularly valuable where noninvasive, high‐resolution visualization of pathological changes is crucial for early detection and monitoring.^[^ ^117^, ^118^, ^119^, ^120^, ^121^, ^122^ ^]^ Additionally, their inherent ultrasmall size enables renal clearance and facilitates passive targeting by leveraging injury‐induced blood–brain barrier permeability, allowing efficient circulation and accumulation at the disease site. Thus, clusters with tunable electronic structure and optical properties at the atomic level offer a unique combination of deep‐tissue penetration, superior spatial resolution, and minimal background autofluorescence, positioning them as a programmable platform for bioimaging.^[^ ^82^, ^86^, ^123^, ^124^, ^125^ ^]^

#### 4.2.1 Vascular Imaging

Brain injury, including traumatic brain injury and stroke, initiates a complex pathological cascade involving cerebral oedema, hemorrhage, and blood–brain barrier (BBB) disruption.^[^ ^61^, ^126^, ^127^ ^]^ These events lead to impaired hemodynamics and secondary neural damage. However, noninvasively capturing the real‐time dynamics of these vascular abnormalities with high spatiotemporal resolution remains a significant challenge, hindering precise diagnosis and evaluation. The emergence of NIR‐II fluorescence imaging has substantially improved deep‐tissue visualization. Atomically precise metal clusters have shown remarkable NIR‐II penetration across tissues. Among them, Ag clusters enabled clear vascular imaging through 2.4 mm of muscle.^[^ ^53^ ^]^ Au clusters achieved imaging depths up to 6.1 mm in kidney tissues, while NIR‐II visualization in tumors, lymph, and blood vessels reached 3–5 cm.^[^ ^87^, ^128^, ^129^ ^]^ This optical window provides an ideal platform for noninvasive monitoring of cerebrovascular networks and hemodynamic changes in vivo.

**Figure 8:**
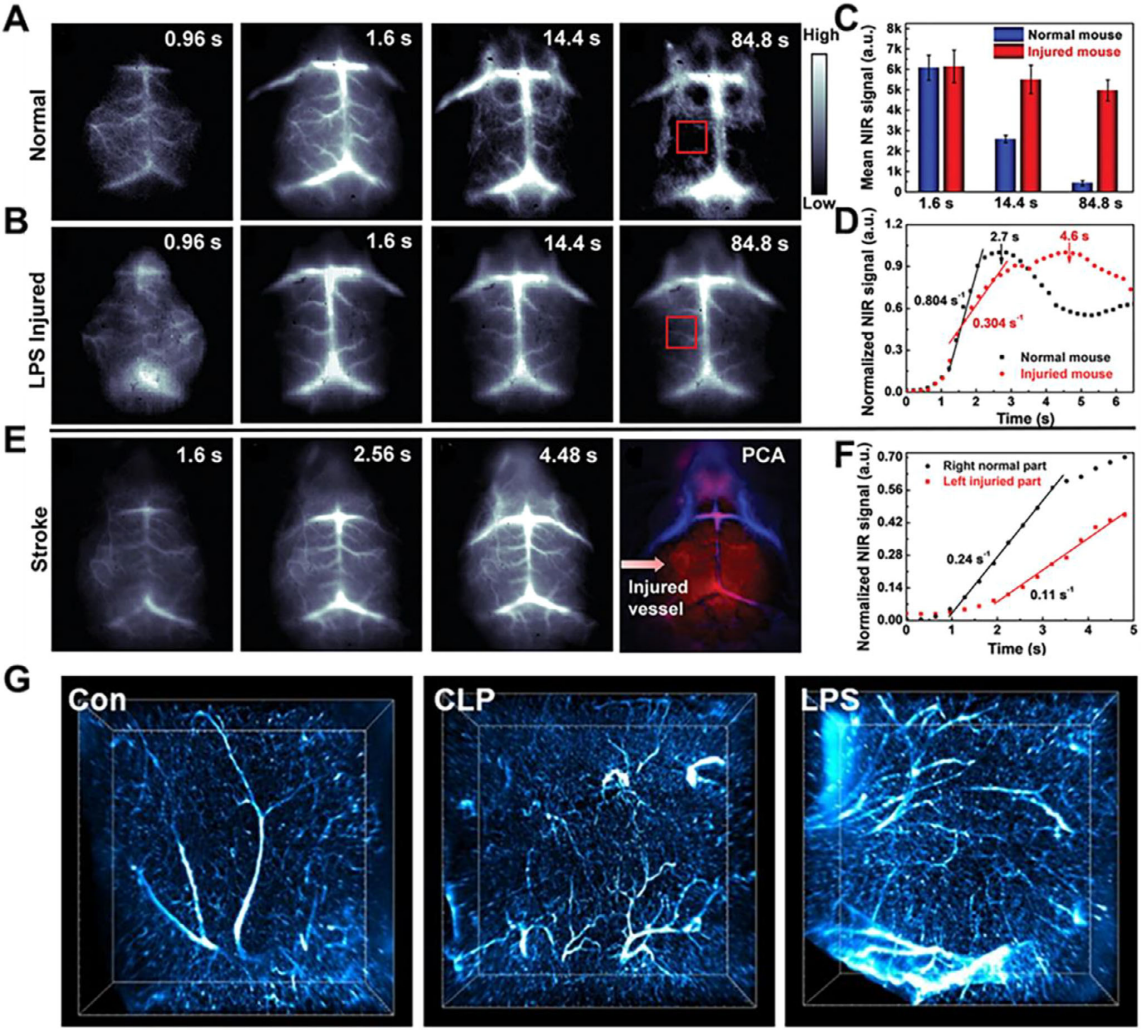
Programmed metal cluster probes for brain injury imaging. A) Dynamic brain imaging of a healthy mouse. B) Dynamic brain imaging of LPS‐injured mouse. C) Quantitative analysis of brain signal in the healthy brain and LPS‐injured brain. D) Blood perfusion of healthy mouse and LPS‐injured mouse. E) Dynamic brain imaging of the stroke mouse, PCA overlaid images with arterial (red) and venous (blue) vessels of the stroke mouse. F) Blood perfusion of the stroke mouse. Reproduced with permission.^[^ ^87^ ^]^ Copyright 2019, John Wiley and Sons. G) 3D volumetric NIR‐II light‐sheet microscopy (LSM) imaging of the sepsis‐induced brain injury. Reproduced with permission.^[^ ^130^ ^]^ Copyright 2025, American Chemical Society.

To address this, gold clusters were programmed as dynamic NIR‐II vascular probes. For instance, Liu et al. employed GSH‐protected gold clusters and Cu‐doped gold clusters for vascular imaging. This manipulation of the atomic‐level doping and surface chemistry programmed clusters into highly sensitive and bright probes of vascular integrity and perfusion kinetics. In vivo imaging studies validated this programming approach. Following intravenous administration in healthy mice, clusters generated high‐resolution angiograms with complete arteriovenous delineation within 1.6 s, followed by rapid clearance with signal disappearance by 84.8 s, establishing a normal hemodynamic baseline. In contrast, lipopolysaccharide (LPS)‐induced neuroinflammatory and ischemic stroke models revealed different kinetic profiles, with probes exhibiting profoundly sustained signal retention due to extravasation through the compromised BBB and impaired clearance mechanisms. Quantitative analysis demonstrated significantly reduced perfusion rates in affected regions (0.11 s^−1^) compared to unaffected areas (0.24 s^−1^), providing measurable parameters for injury severity (**Figure** **8**A–F).^[^ ^87^ ^]^ Additionally, Li et al. employed atomic engineering to develop Au_21_Ni_1_ clusters for detailed visualization of microstructural lesions and disease progression (Figure 8G).^[^ ^130^ ^]^ 3D visualization revealed that in sepsis‐induced brain injury, the brain tissue was affected by inflammation, with vessels exhibiting significant swelling and dilation, leading to the aggregation of clusters within the vasculature. Concurrently, stimulated by the inflammatory response, local brain tissue released growth factors, promoting the proliferation of vascular endothelial cells and the formation of new blood vessels. This process represents a physiological response of the body to repair damage, yet excessive proliferation may contribute to disease progression. These findings establish that cluster‐based imaging can detect subtle cerebrovascular pathologies through quantitative mapping of hemodynamic retention patterns, offering a powerful tool for diagnosing, stratifying, and monitoring brain injury progression.

#### 4.2.2 Alzheimer's Disease Detection

The pathological process of AD begins years before clinical symptoms appear, creating a critical diagnostic window where intervention would be most effective.^[^ ^131^ ^]^ However, current imaging techniques primarily detect advanced pathology, such as Aβ plaques, leaving a significant unmet need for probes capable of identifying upstream molecular events during the preclinical phase.^[^ ^132^, ^133^, ^134^ ^]^

**Figure 9:**
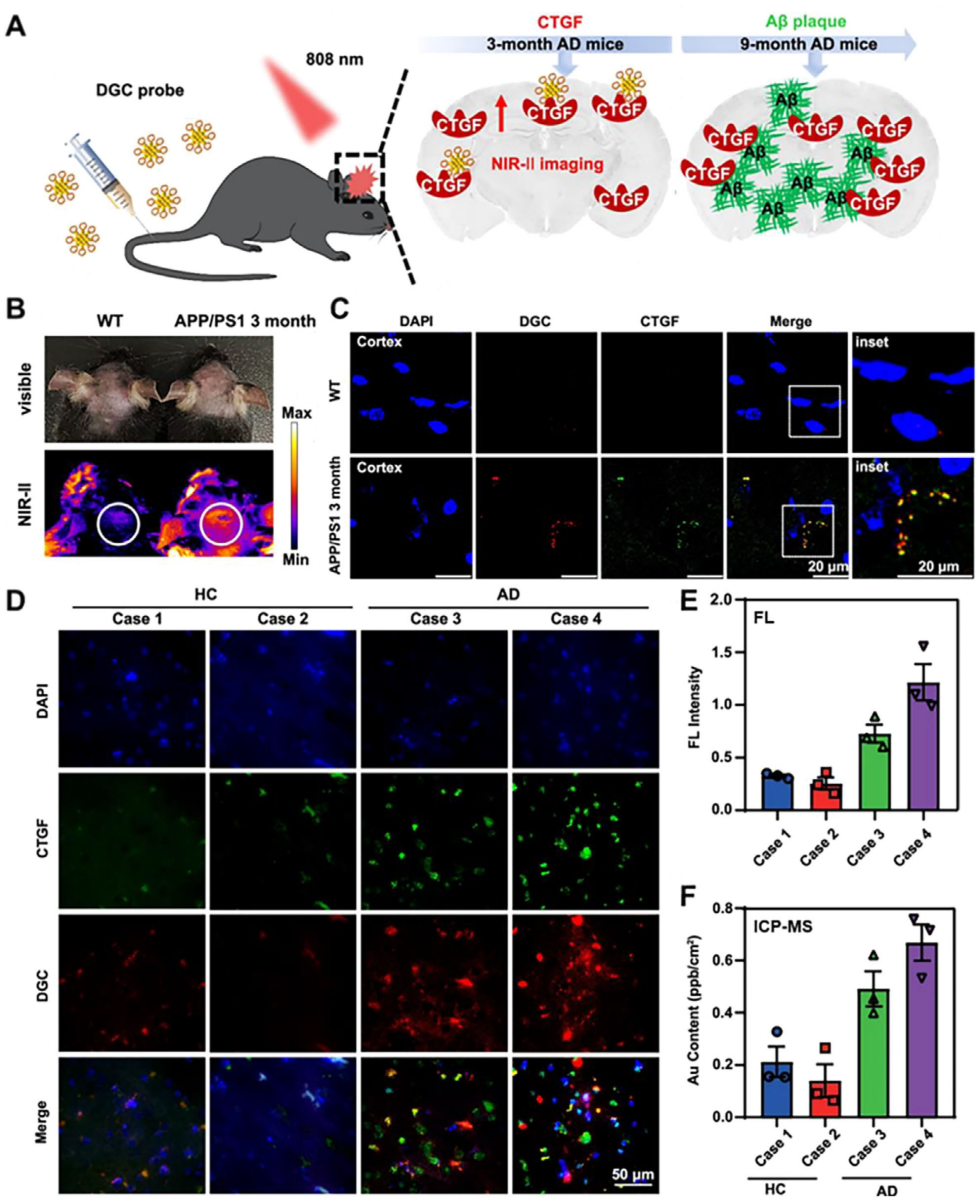
Programmed cluster probes for early Alzheimer's disease detection. A) Design of a connective tissue growth factor (CTGF)‐targeting clusterzyme probe (DCG) for NIR‐II imaging and early diagnosis of AD. B,C) Validation in APP/PS1 transgenic mice that the CTGF‐programmed probe achieves specific accumulation and high signal‐to‐noise ratio imaging in disease‐relevant brain regions. D) The fluorescence imaging of brain sections from an early stage of an AD patient (AD) and a healthy control (HC) after costaining with DGC and CTGF. E) The fluorescence (FL) intensity of DGC. F) ICP‐MS analysis of DGC (Au) content per cm^2^. Reproduced under terms of the CC‐BY license.^[^ ^135^ ^]^ Copyright 2024, Cao Lu et al., published by Springer Nature.

To address this challenge, clusters were programmed as molecular‐specific diagnostic agents through rational surface engineering. A pioneering example is the connective tissue growth factor (CTGF)‐targeting cluster probe (DCG), where the cluster surface was functionally programmed with CTGF‐specific targeting moieties (**Figure** **9**).^[^ ^135^ ^]^ This design leveraged the overexpression of connective tissue growth factor (CTGF) as an early biomarker in AD pathogenesis, programming the clusters for active targeting of presymptomatic pathology. The design also maintained the inherent NIR‐II optical advantages of the cluster for deep‐tissue imaging. This targeted programming strategy demonstrated remarkable success in both animal models and human tissue studies. In APP/PS1 transgenic mice, the CTGF‐programmed clusters showed significantly enhanced accumulation in disease‐relevant brain regions, with NIR‐II fluorescence intensity substantially higher than in wild‐type controls, indicating specific binding to early AD pathology. Crucially, validation in human postmortem brain tissues revealed strong colocalization with CTGF expression and significantly higher accumulation in Thal stage 1 AD samples compared to healthy controls, as confirmed by both fluorescence imaging and inductively coupled plasma‐mass spectrometry (ICP‐MS) quantification.^[^ ^135^ ^]^ The programmability of clusters, encompassing both their intrinsic optical properties and extrinsic targeting capabilities, establishes them as a transformative platform for disease detection. This flexibility allows for the design of probes that meet distinct diagnostic needs, from assessing vascular injury to enabling the early detection of neurodegenerative diseases, further monitoring the pathological progression.

### 4.3 Cluster‐Based Neural Electrodes for Brain–Computer Interfaces

The integration of cluster with neural interfaces represents a paradigm shift in bioelectronics, demonstrating how programmable biocatalysis can overcome fundamental challenges in neural recording and stimulation.^[^ ^136^, ^137^, ^138^, ^139^, ^140^ ^]^ Conventional neural electrodes face persistent limitations in signal fidelity and chronic biocompatibility due to interfacial mismatch and foreign body responses. Clusters, with their atomically defined structures and tunable multiple enzyme‐like activities, offer a rational design to these challenges by creating bioactive interfaces that seamlessly integrate with neural tissue.^[^ ^140^, ^141^ ^]^

**Figure 10:**
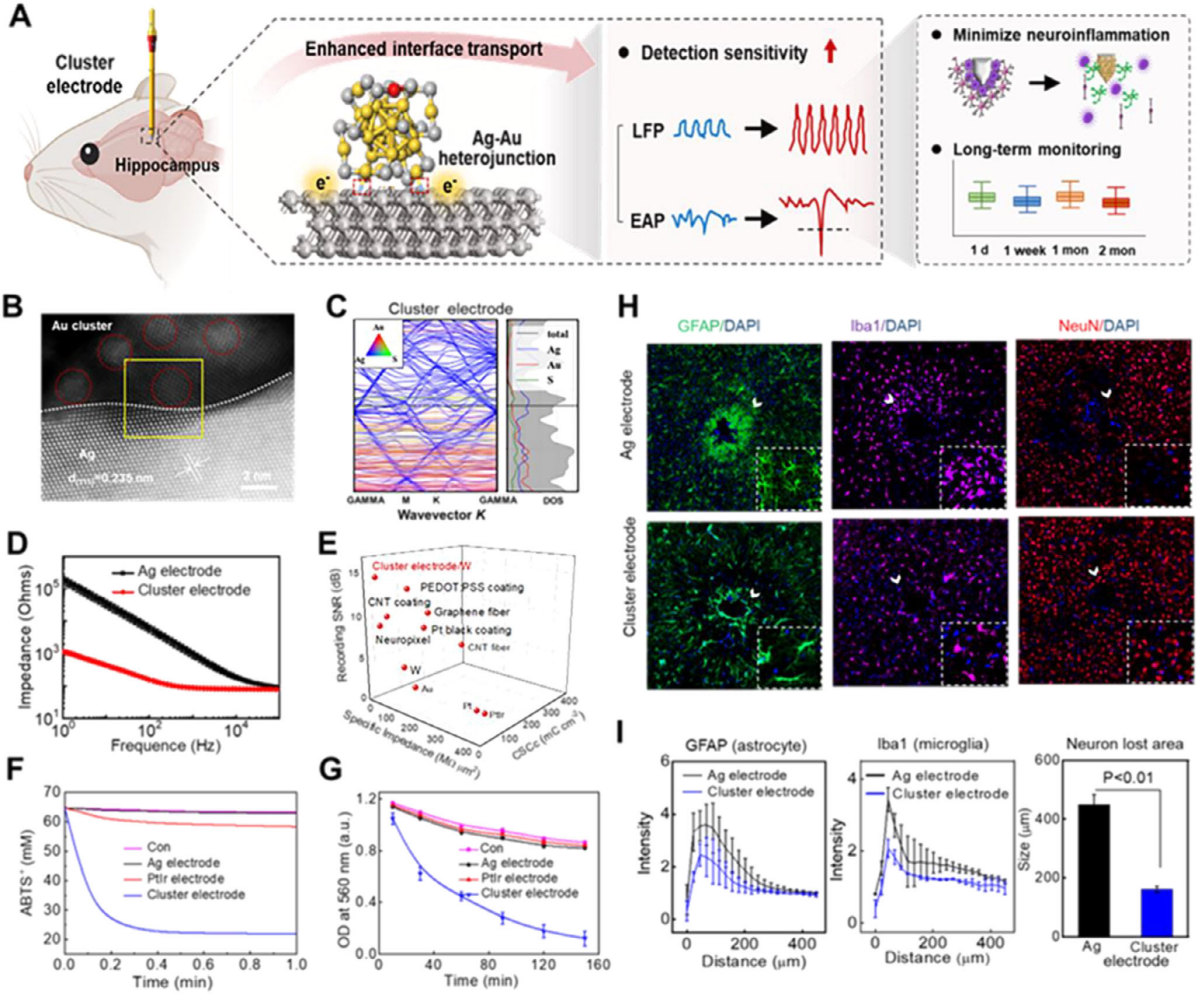
Performance of programmed cluster neural electrode. A) Schematic illustration of the performance for the cluster electrode. Atomically precise Au clusters on an Ag electrode form a heterogeneous Au–Ag interface that programs enhanced charge transport, sensitivity, biocompatibility. B) HAADF‐STEM image showing Au–Ag bonds and Ag dislocations at the cluster interface. The Ag atomic layer at the interface exhibited a lattice stripe of 0.235 nm corresponding to the (111) plane of the Ag crystal. C) Band structure and density of states of the cluster electrode, indicating significant contributions from Au and S atoms. The impedance of the nanozyme electrodes was significantly lower than that of the Ag electrodes in the frequency range of 1 Hz–100 kHz. E) Comparison of the specific impedance at 1 kHz, CSCc, single‐unit SNR of cluster electrodes with the state‐of‐the‐art neural interfacing electrodes reported in the literature. D) Impedance spectra and CV curves for cluster electrode. E) Comparison of the specific impedance at 1 kHz, CSCc, single‐unit SNR of cluster electrodes with the state‐of‐the‐art neural interfacing electrodes reported in the literature. F,G) Superior antioxidant and SOD‐like activity, showing substantially higher enzymatic activity on cluster surfaces relative to Ag and PtIr electrodes. H) Immunofluorescence staining of tissue responses following 2‐month implantation of Ag electrodes and cluster electrodes for astrocytes (green, microglia (purple, neurons (red). Scale bar, 100 µm. I) Normalized fluorescence intensities for nanozyme and Ag electrodes. Reproduced with permission.^[^ ^139^ ^]^ Copyright 2023, John Wiley and Sons.

The functionalization of neural electrodes with a cluster creates heterogeneous interfaces that fundamentally enhance charge transfer properties. In a seminal demonstration, Zhang et al. integrated atomically precise gold clusters onto Ag electrodes, forming a programmed Au–Ag interface that can achieve high‐speed charge transfer and enhance detection sensitivity (**Figure** **10**A). The emerging cluster neural electrodes exhibited unique enzyme‐mimicking catalytic activity, which can reduce neuroinflammation and enhance biocompatibility. To elucidate the structural origin of these enhanced electrochemical and biological properties, high‐angle annular dark‐field scanning transmission electron microscopy (HAADF‐STEM) revealed the atomic‐level structure of the cluster electrode interface, which showed Au–Ag bonds and Ag dislocations that generated highly active sites and enhanced electrode performance (Figure 10B). Consistent with these structural findings, analysis of the band structure and density of states indicated that Au and S atoms contributed significantly to the electronic states, providing a broader range of conduction bands for free electrons and thereby improving signal transduction efficiency (Figure 10C). This electronic modulation explained the underlying mechanism responsible for fast electron transport properties, as the nanostructure exposes sufficient sites for ion transmission and transfer, resulting in the enhancement of signal transduction. As a result, the cluster electrode exhibited a 12‐fold reduction in electrochemical impedance at 1 kHz and a 26‐fold increase in charge storage capacity compared to Ag electrodes (Figure 10D,E).^[^ ^139^ ^]^ Compared with state‐of‐the‐art neural interface materials, such as poly (3,4‐ethylenedioxythiophene)‐poly (styrenesulfonate) (PEDOT), Pt black, graphene, and carbon nanotube, cluster electrodes exhibit superior or comparable electrochemical performance in terms of charge storage capacity (CSC), impedance, and signal‐to‐noise ratio (SNR) (Figure 10E). Notably, the impedance of the cluster electrodes (16.6 MΩ µm^2^) was lower than that of the Graphene fiber (28.4 MΩ µm^2^) and the PEDOT:polystyrene sulfonic acid (PSS) coating (18 MΩ µm^2^). Moreover, the cluster electrode achieved a higher SNR of 14.7 dB, which was 4.5‐ and 2‐fold than that of the CNT fiber and Pt black coating electrode. The enhanced electrochemical performance stemmed from the precisely controlled electronic structure of the cluster, which facilitated efficient electron transfer across the electrode‐tissue interface. Complementary materials innovations, including nanoporous graphene microelectrodes with high charge injection capacity (5 mC cm^−2^) and metal chalcogenide nanotube arrays with exceptional electrocatalytic activity across broad pH ranges, further illustrated how nanomaterial programming enables next‐generation neural interfaces.^[^ ^142^, ^143^ ^]^

The most distinctive advantage of cluster interfaces lies in their programmable biochemical functionality.^[^ ^140^, ^144^ ^]^ Unlike conventional bioinert coatings, cluster electrodes actively suppress neuroinflammation and oxidative stress through inherent multienzyme activities.^[^ ^145^ ^]^ Experimental measurements revealed that cluster surfaces exhibited antioxidant capacities 110.6 and 12.6 times greater than Ag and PtIr electrodes, respectively, along with superoxide dismutase‐like activity 20 times higher than conventional materials (Figure 10F,G).^[^ ^139^ ^]^ These enzyme‐mimicking catalytic activities effectively suppressed ROS accumulation, thereby alleviating oxidative stress at the tissue interface. Such programmable catalytic functionality translated directly to improved biological integration, alleviated oxidative stress at the tissue interface. Immunofluorescence analyses demonstrated a 1.5‐fold reduction in glial scar formation, a 1.6‐fold decrease in activated microglia, and significantly mitigated neuronal death around implant sites (Figure 10H,I).^[^ ^139^ ^]^ Moreover, cluster integration addressed the critical challenge of mechanical mismatch between rigid electrodes and soft brain tissue, leading to improved mechanical compliance and reduced local strain during implantation.^[^ ^146^, ^147^, ^148^ ^]^

**Figure 11:**
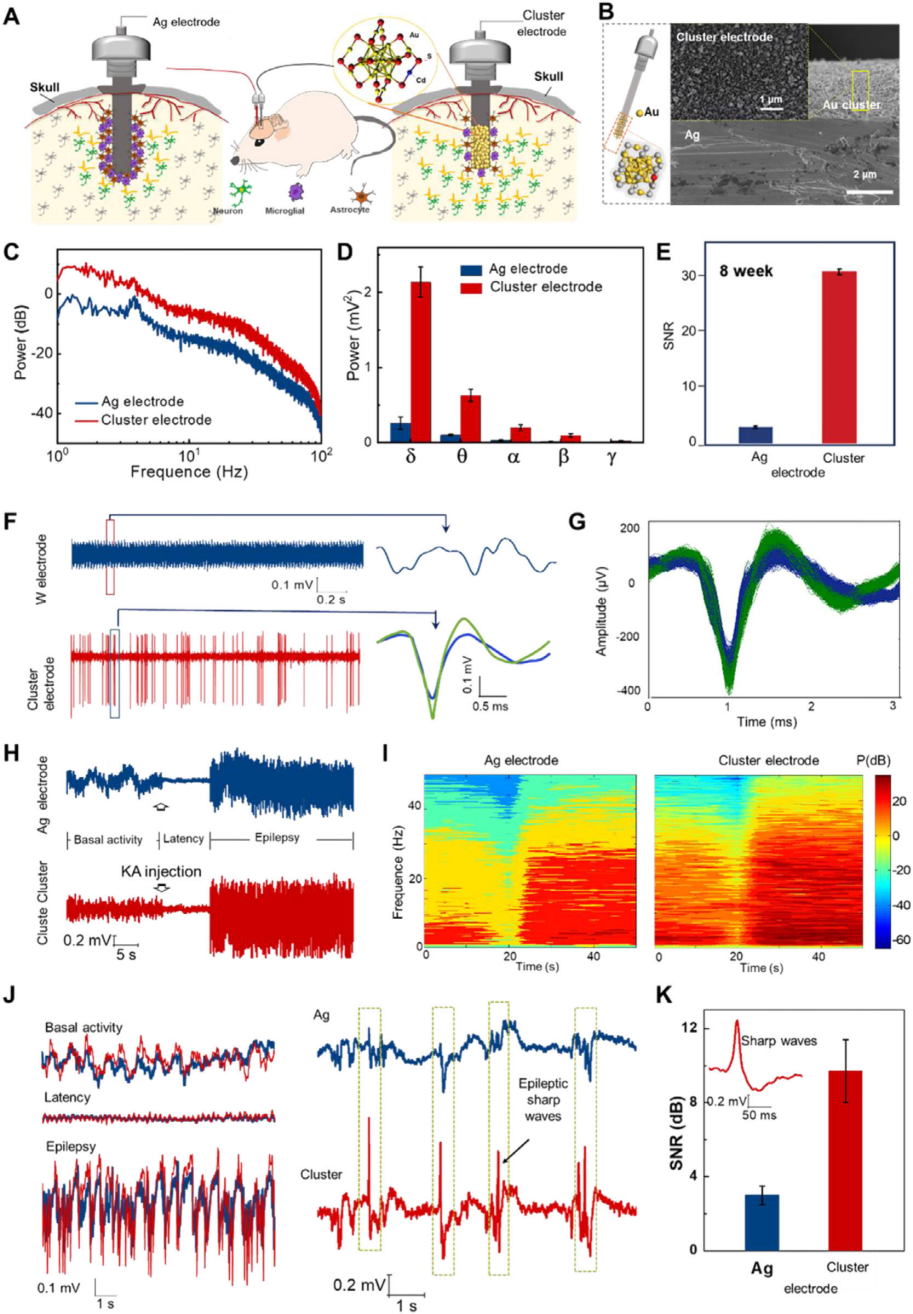
Detection sensitivity of the electrophysiological signal with a cluster electrode. A) A schematic drawing of the implanted nanozyme electrodes in the hippocampus CA3. B) Cluster electrode used for signal recording. C,D) The power spectra analysis and quantitative analysis in the low‐frequency band demonstrate significant amplification compared with Ag electrodes. E) SNR after 8 weeks of implanted nanozyme electrodes, showing improved recording fidelity with nanozyme electrodes. F,G) Representative acute recordings of high‐frequency neural activity and isolated single‐unit spikes. H,I) Detection of pathological spike activity during epileptic seizures from acute seizure rat models. J,K) Enlarged view of baseline, latency, epileptiform periods; sharp wave discharges detected at seizure onset by the cluster electrode demonstrating its high temporal resolution and sensitivity. Reproduced with permission.^[^ ^139^ ^]^ Copyright 2023, John Wiley and Sons.

The combination of enhanced electrochemical performance and active biocompatibility culminates in unprecedented neural recording capabilities.^[^ ^139^, ^149^ ^]^ Cluster electrodes achieved remarkable signal‐to‐noise ratio improvements, enabling capture of weak neural signals previously undetectable with conventional interfaces. In rodent models, these cluster electrodes exhibited a pronounced increase in δ rhythm amplitude in local field potential recordings from hippocampus CA3 (**Figure** **11**A,B). Spectral analyses further confirmed a 14‐fold enhancement in the low‐frequency δ band, highlighting the superior sensitivity of cluster electrodes in detecting subtle neural oscillations (Figure 11C,D). Moreover, the SNR of cluster electrode maintained a 6.2‐fold higher than that of unmodified electrodes over 8‐week implantation periods, demonstrating long‐term stability and reliability for chronic recordings (Figure 11E). Furthermore, the cluster electrodes enabled the detection and isolation of single‐neuron spikes with high fidelity, allowing for detailed decoding of neural activity and potential applications in advanced brain–computer interface (Figure 11F,G). The enhanced sensitivity derived from the clustered architecture was further demonstrated in epilepsy models, where cluster electrodes captured neuron firing activities with high fidelity across all seizure phases (Figure 11H,I). Especially, cluster electrodes can detect pathological signals that failed to be captured by traditional electrodes during epileptic seizures (Figure 11J,K). The high neural activity of the cluster electrode was 6.0, 1.7, and 2.1 times higher than that of the Ag electrodes in the basal, latency, and epilepsy periods, respectively. Cluster electrodes can considerably improve the SNR of seizures in acute epileptic rats and are expected to achieve precise localization of seizure foci in clinical settings.

The integration of clusters with neural interfaces exemplifies the power of programmable nanomaterials to transform biomedical devices. By rationally designing interfaces that combine enhanced charge transfer, mechanical compliance, and active biochemical regulation, cluster‐based neural platforms overcome fundamental limitations of conventional electrodes, opening new possibilities for precise neural interrogation and therapeutic modulation in complex neurological conditions.

## 5 Conclusion and Outlook

**Figure 12:**
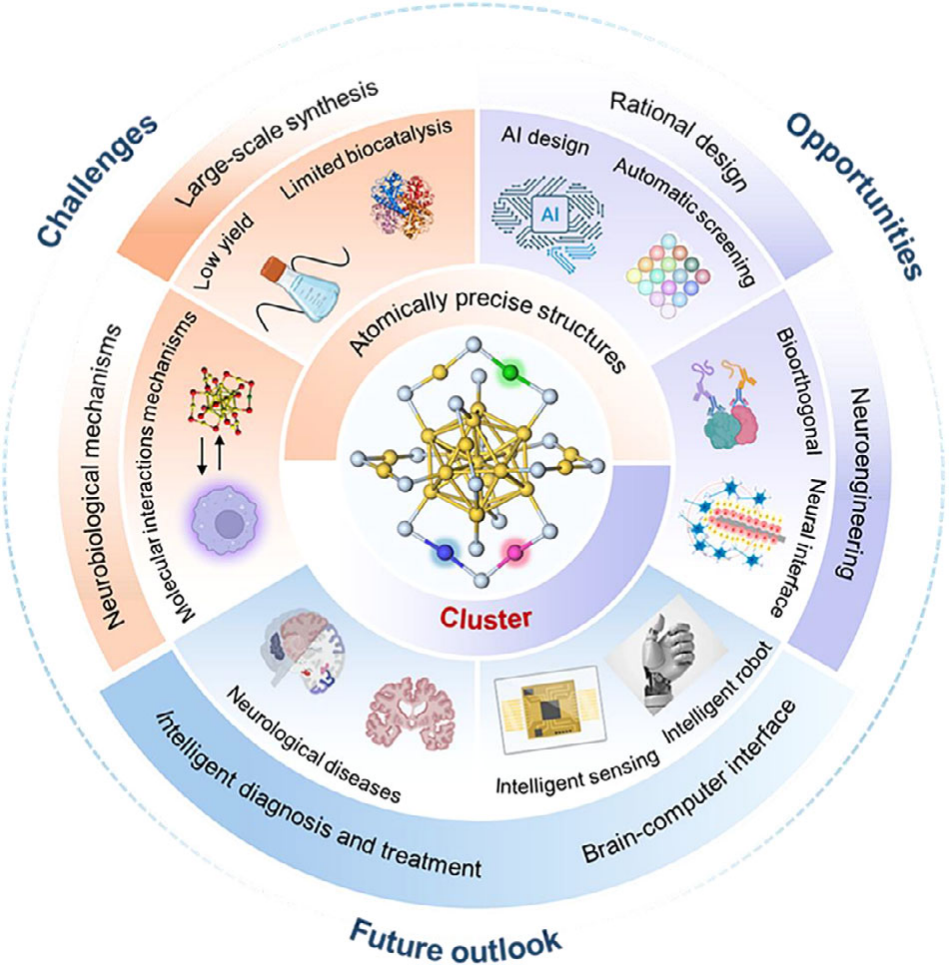
Challenges and outlook of clusters as an artificial enzyme platform. The diagram summarizes the key challenges, such as scalable synthesis and neurological mechanisms, opportunities, such as AI‐driven and high‐throughput design, the outlook of programmable clusters for precise diagnosis and treatment and brain–computer interfaces.

Despite these promising advances, several challenges must be addressed to realize the potential of clusters (**Figure** **12**).^[^ ^150^ ^]^ First, scalable and reproducible synthesis remains a bottleneck. Atomically precise metal clusters are sensitive to reaction conditions, where minor environmental variations can induce size heterogeneity or impurities. Complex ligands, sluggish kinetics, and multistep purification procedures constrain throughput, while their narrow thermodynamic stability window readily leads to larger‐scale aggregation or decomposition. Although advanced techniques like flow chemistry show promise for improving reproducibility, achieving gram‐scale production of structurally uniform clusters demands further innovation.^[^ ^26^, ^151^, ^152^, ^153^ ^]^ For instance, while Ag_28_Pt_1_ bimetallic clusters exhibit high antioxidant activity, the precise control of metal stacking sequences and ligand protection strategies remains challenging.^[^ ^53^ ^]^ Second, the functional repertoire of clusters requires expansion beyond classical antioxidant activities. The potential for catalyzing reactions involving atypical signaling molecules, such as hydrolase, kinase, and dehydrogenase, remains underexplored.^[^ ^154^, ^155^ ^]^ Incorporating nontraditional metal elements, such as Ir, Pd, or constructing hybrid structures could diversify their catalytic performance.^[^ ^156^ ^]^ For example, designing clusters with cytochrome c reductase activity or lipase‐like activity may enable the detection and regulation of metabolites associated with metabolic disorders.^[^ ^154^, ^157^ ^]^ Third, neurological mechanisms need to be further clarified. While state‐of‐the‐art studies focus on the enzyme‐like activity of the clusters in diverse diseases, the precise molecular interactions between clusters and neural tissues, along with the ensuing cascade of biochemical responses, remain largely unclear.^[^ ^101^, ^158^, ^159^, ^160^, ^161^, ^162^, ^163^, ^164^, ^165^, ^166^, ^167^ ^]^

The integration of artificial intelligence (AI) and quantum chemical calculations is poised to play a crucial role in overcoming these challenges. A transformative shift from forward screening to inverse design is emerging. This approach starts with desired target functions, such as exceptional SOD‐like activity, specific NIR II emission peaks, or selective catalysis of particular metabolites, as input parameters. Artificial intelligence models then generate novel atomic combinations and structural blueprints, each optimized to achieve the preset function. This approach dramatically enhances the autonomy and predictability of material design, promising to shorten the discovery cycle for novel cluster enzymes from years to weeks while exploring vast chemical spaces that are inaccessible or counterintuitive to human researchers. The realization of this vision, however, faces significant challenges. Its success is critically dependent on large‐scale, high‐quality experimental datasets for training, which remain scarce for atomically precise clusters. Furthermore, key hurdles include the physical interpretability of the models and the experimental synthesizability of the predicted structures. Despite these challenges, pioneering studies demonstrate its profound potential. For instance, deep learning has been employed to inversely design 3D chiral clusters with strong chiroptical responses.^[^ ^61^ ^]^ Similarly, Hata et al. used a deep learning framework to inversely predict the catalytic activity and selectivity of clusters by integrating structural characterization.^[^ ^168^ ^]^ These works lay the groundwork for the ultimate goal of function‐driven rational design. Looking ahead, integrating atomically precise cluster structures into larger‐scale responsive biomedical systems will emerge as a highly promising research frontier. Reverse engineering can guide the construction of intelligent systems, where cluster structures are enhanced through embedding within smart architectures or utilizing precision carriers to deliver programmable catalytic activity. The surrounding matrix then facilitates targeted positioning, activation regulation, or signal transduction. This strategy is complemented by bio‐orthogonal chemistry for spatiotemporal control and by the development of clusters responsive to magnetic, acoustic, or optical stimuli for deep‐tissue neuromodulation.^[^ ^169^, ^170^ ^]^ For instance, the charge‐state‐dependent magnetic behavior of Au_25_(SR)_18_ and the multishell electronic organization of Au_133_(SR)_52_ indicate that atomic‐level modulation of spin–orbit interactions may be exploited to generate magneto‐electric coupling at the nanoscale. These clusters can be rendered water‐soluble through established ligand exchange strategies, and these properties position atomically precise gold clusters as promising building blocks for future magneto‐electric transducers aimed at wireless, deep‐tissue neuromodulation.^[^ ^171^, ^172^, ^173^ ^]^

By harnessing their programmable biocatalytic, optical, and electronic properties, these atomic‐level clusters offer unprecedented opportunities to achieve intelligent diagnosis, precise treatment, and efficient brain–computer interfacing. The ability to rationally design clusters for specific neural pathways or pathological markers will open pathways to personalized therapeutic strategies and closed‐loop neuroelectronic systems. Ultimately, this programmability positions clusters as a foundational tool for interfacing with the brain, not only treating disorders but also augmenting neural function and enabling direct communication between biological and digital systems.

## Conflict of Interest

The authors declare no conflict of interest.

## Author Contributions

S.S., D.L., S.Z., and Y.W. contributed to writing the first draft of the paper, visualization, writing–review and editing. H.W. helped in writing the first draft of the paper. Z.Z. and X.‐D.Z. assisted in conceptualization, writing the first draft of the paper, writing–review and editing and helped in funding acquisition.
